# B cell/adipocyte crosstalk amplifies MCP-1 secretion, M2-like macrophage accumulation, and polarization in gut-associated, inflamed adipose tissue

**DOI:** 10.1093/ecco-jcc/jjag182

**Published:** 2026-11-07

**Authors:** Marie Christine Goess, Julia M Heinze, Julian Hofmann, Annalisa Buck, Iana Gadjalova, Marie-Christin Weber, Sandra Ring, Joerg M Buescher, Matteo Villa, Philipp-Alexander Neumann, Jürgen Ruland, Selina J Keppler

**Affiliations:** Institute of Laboratory Medicine, School of Medicine and Health, TUM University Hospital, Munich, Germany; TranslaTUM, Central Institute for Translational Cancer Research, Munich, Germany; Institute of Laboratory Medicine, School of Medicine and Health, TUM University Hospital, Munich, Germany; TranslaTUM, Central Institute for Translational Cancer Research, Munich, Germany; Turku Bioscience Centre, University of Turku and Åbo Akademi University, Turku, Finland; Department of Surgery, Technical University of Munich, School of Medicine and Health, Munich, Germany; Institute for Advanced Study, Technical University of Munich, Munich, Germany; Institute of Laboratory Medicine, School of Medicine and Health, TUM University Hospital, Munich, Germany; TranslaTUM, Central Institute for Translational Cancer Research, Munich, Germany; Department of Surgery, Technical University of Munich, School of Medicine and Health, Munich, Germany; Institute of Laboratory Medicine, School of Medicine and Health, TUM University Hospital, Munich, Germany; TranslaTUM, Central Institute for Translational Cancer Research, Munich, Germany; Max Planck Institute for Immunobiology and Epigenetics, Freiburg 79108, Germany; Division of Rheumatology and Immunology, Medical University Graz, Graz, Austria; Department of Surgery, Technical University of Munich, School of Medicine and Health, Munich, Germany; Institute for Advanced Study, Technical University of Munich, Munich, Germany; Institute of Laboratory Medicine, School of Medicine and Health, TUM University Hospital, Munich, Germany; TranslaTUM, Central Institute for Translational Cancer Research, Munich, Germany; German Cancer Consortium (DKTK), partner site Munich, a partnership between DKFZ, TUM University Hospital, Munich, Germany; German Center for Infection Research (DZIF), partner side Munich, Munich, Germany; German Center for Child and Adolescent Health (DZKJ), partner side Munich, Munich, Germany; Institute of Laboratory Medicine, School of Medicine and Health, TUM University Hospital, Munich, Germany; TranslaTUM, Central Institute for Translational Cancer Research, Munich, Germany; Division of Rheumatology and Immunology, Medical University Graz, Graz, Austria

**Keywords:** IBD, B cells, adipose tissue

## Abstract

**Background and Aims:**

During Crohn’s disease (CD), hyperplasia of the mesenteric white adipose tissue (WAT), called creeping fat (CrF), is associated with the accumulation of immune cells. Here we investigated the impact of B cell/adipocyte crosstalk on immune cell recruitment and inflammation within inflammatory bowel disease-related adipose tissue.

**Methods:**

We used flow cytometry and *in vitro* 3D culture systems of biopsies from CrF of CD patients as well as a mouse model of intestinal inflammation, *in vitro* co-cultures, mass spectrometry, and single-cell RNA sequencing to evaluate the impact of B cell/adipocyte interplay on monocyte chemoattractant protein-1 (MCP-1) production and macrophage polarization.

**Results:**

Employing primary human and mouse 3D adipocyte cultures, we observed that adipocytes from CD patients displayed a persistent inflammatory phenotype even after prolonged time in culture, a cytokine secretion profile also recapitulated via lipopolysaccharide stimulation. Analyzing CrF of CD patients as well as WAT of a mouse model of intestinal inflammation, we found an accumulation of B cells. B cell/adipocyte co-cultures led to elevated secretion of MCP-1 by primary adipocytes, facilitating macrophage migration. Consequently, co-transfer of B cells with T cells increased T cell activation and M2-like macrophage accumulation in inflamed mouse WAT. Mass spectrometry and single-cell RNA sequencing analyses suggested that the B cell/adipocyte crosstalk establishes a microenvironment that may promote M2-like macrophage polarization.

**Conclusion:**

B cell/adipocyte interactions in CD-associated inflamed adipose tissue increase MCP-1 and lactate, promoting recruitment and M2-like polarization of macrophages, likely amplifying local WAT inflammation.

## Graphical abstract

**Graphical Abstract:**
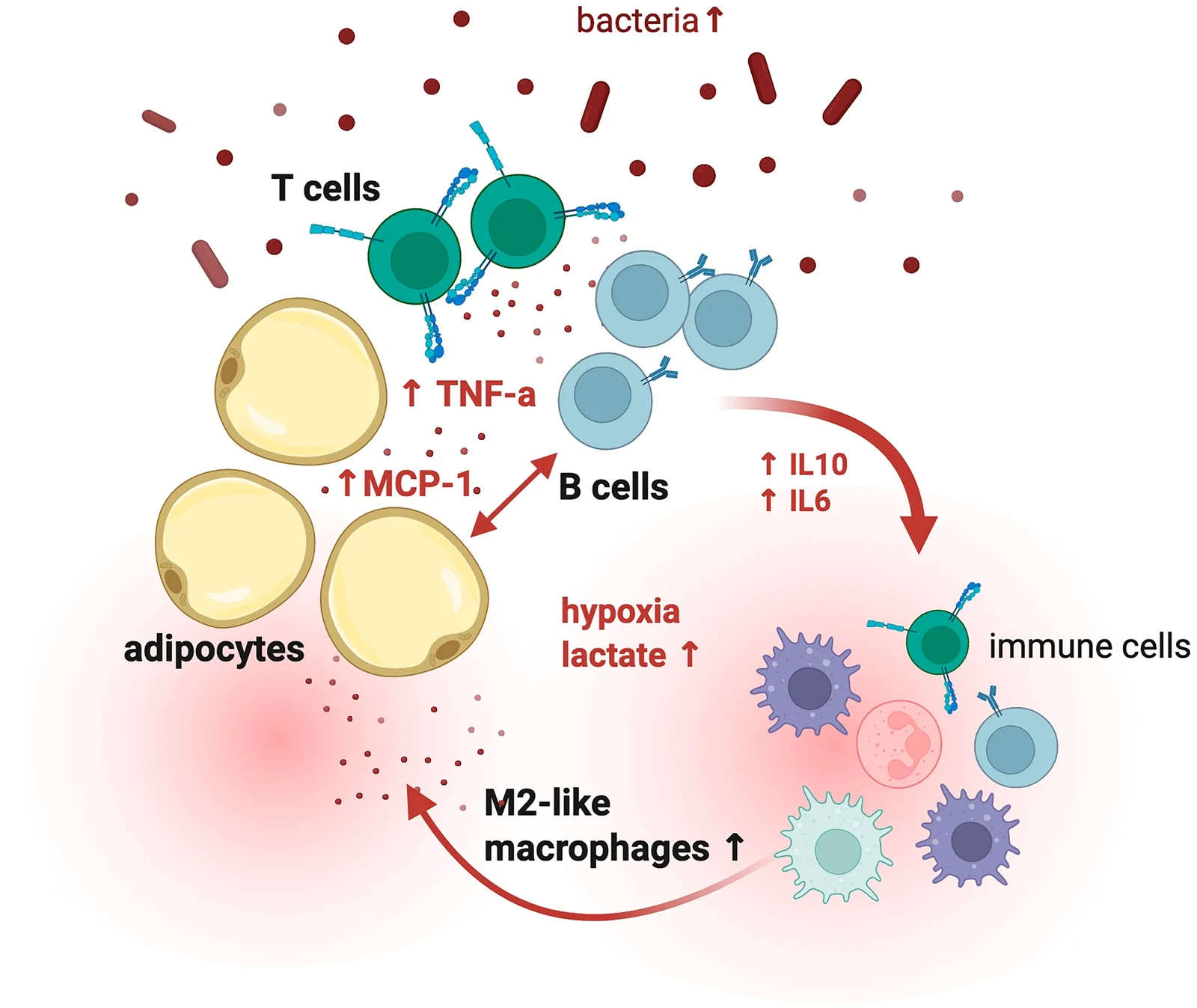

## 1. Introduction

Inflammatory bowel disease (IBD), including Crohn’s disease (CD) and ulcerative colitis (UC), is a group of immune-mediated disorders of the intestine. During IBD, a barrier breach of the intestinal epithelium results in microbial translocation into the surrounding tissue, including the mesenteric white adipose tissue (MAT),^1^ resulting in inflammation. During CD, the inflamed MAT displays adipocyte hyperplasia^2^ eventually leading to fat-wrapping around the intestine (so-called “creeping fat” [CrF]^3^) CrF is an extra-intestinal inflammation of CD, which is thought to restrict bacterial dissemination and the spread of inflammation, but also correlates with complications, such as intestinal fibrosis and stricture formation.^4^ We and others have previously shown that adipocytes in CrF are smaller^5^^,^^6^ and seem to be metabolically dysfunctional, shifting from fatty acid synthesis to glycolysis and showing signs of hypoxia.^7^

Inflammatory adipose tissue during CD is also characterized by the production of pro-inflammatory cytokines such as interleukin-6 (IL-6), Tumor necrosis factor alpha (TNF-α), IL-1, and monocyte chemoattractant protein-1 (MCP-1, also known as C-C chemokine ligand 2 [CCL2]).^7^^,^^8^ This local release of pro-inflammatory cytokines might lead to the recruitment and activation of immune cells. CrF of CD patients has been demonstrated to favor the differentiation and accumulation of pro-fibrotic, M2-like, CD206-expressing macrophages.^1^^,^^9^ In addition, studies of CD patients report immune cell aggregates resembling tertiary lymphoid organs around lymphatic vessel of the MAT, adjacent to areas of intestinal inflammation.^9-11^ A recent transcriptomics study of CrF identifies markers of B cells, T cells, as well as plasma cells associated with CD.^1^^,^^12^

B cells might influence MAT inflammation through antibody and cytokine production or interaction with immune or stroma cells. While the phenotype and function of infiltrating immune cells during diet-induced or age-associated obesity has been studied extensively,^13-16^ little is known about the phenotype and function of WAT infiltrating B cells during inflammatory, non-obese conditions, such as during IBD.

Here, we investigated how interactions between B cells and inflamed adipocytes affect immune cell recruitment and inflammation in IBD-related adipose tissue. We established primary human and mouse 3D adipocyte culture systems to study adipocyte inflammation and adipocyte/B cell interaction *in vitro*. We found that adipocytes from CrF of CD patients display a persistent inflammatory phenotype characterized by persistent cytokine secretion *in vitro* without further stimulation, which could be recapitulated by lipopolysaccharide (LPS)-stimulation of human and mouse primary adipocytes *in vitro*. Our results further suggest that WAT infiltrating B cells enhanced MCP-1 secretion by adipocytes, resulting in increased macrophage migration. Consequently, co-transferring B cells with T cells in an acute model of colitis enhanced M2-like macrophage accumulation in inflamed mouse WAT. Mechanistically, mass spectrometry from *in vitro* co-cultures as well as single-cell RNA sequencing analysis from human and mouse datasets suggest that the B cell/adipocyte crosstalk provided a lactate-rich, inflammatory environment which supported M2-like macrophage polarization in inflamed WAT. Taken together, our results provide evidence for a crucial role of B cell/adipocyte crosstalk to IBD-associated adipose tissue inflammation.

## 2. Methods

### 2.1. Study approval

Informed, written consent was obtained from all patients during their pre-operative visit, with prior approval of the local ethics committee of the Faculty of Medicine of the Technical University of Munich, including 2-fold pseudonymization for patient tissue (TUM; #1926/2007, #5428/12, and 2022-297-S22-KH). Fresh mesenteric adipose tissue samples were collected adjacent to inflamed/fibrotic and non-involved bowel segments from CD patients undergoing intestinal resections. Human studies included both male and female participants as documented in Table 1 and Table S1. Tissue sampling was performed in accordance with the regulations of the tissue bank of the TUM and Klinikum rechts der Isar, Munich (MTBIO).

**Table 1.** Summary of patients’ characteristics.

Characteristic		*n* = 17
**Age at time of surgery (years), median [IQR]**		50 [21]
**Age at diagnosis (years), median [IQR]**		24 [5]
**Gender, *n* [%]**	Male Female	10 [59] 7 [41]
**Disease behavior, *n* [%]** **Montreal classification**	B1: Nonstricturing B2: Stricturing B3: Penetrating	0 [0] 17 [100] 0 [0]
**Localization [%]**	Terminal ileum Colon	15 [88] 2 [12]
**Medication at the timepoint of surgery, *n* [%]**	Adalimumab Infliximab Ustekinumab Glucocorticoids None	1 [6] 2 [12] 6 [35] 6 [35] 2 [12]
**BMI, median [IQR]**		23 [2]
**Smoking status *n* [%]**	Smoker Nonsmoker	4 [24] 13 [76]

All experiments involving mice were conducted in compliance with federal and institutional guidelines, and animal protocols were approved by the Government of Upper Bavaria (Regierung von Oberbayern). Our study examined male and female animals, and similar findings are reported for both sexes.

### 2.2. Isolation of human primary cells

MAT and CrF samples were collected by trained surgeons, washed with Ringer’s solution, weighed, and minced. Tissues were enzymatically digested for 1 h at 371 °C in HBSS buffer containing Ca^2+^/Mg^2+^, 10 mM HEPES, 5% FCS, 1% penicillin-streptomycin, 3% BSA, 1 mg/mL collagenase D, 0.5 mg/mL dispase II, and 0.25 mg/mL DNase I. The resulting suspension was filtered through a 100-μm strainer, and lymphocytes were purified through a Percoll (GE Healthcare) gradient (2200 rpm, 20 min, 4 °C, 1 acceleration, 0 deceleration) harvested at the 40%/80% interface and washed in B cell medium (RPMI-1650 + GlutaMax, 10 mM HEPES, 10% FCS, 1% P/S, and 0.05 mM 2-mercaptoethanol).

### 2.3. Flow cytometric analysis

Human and mouse single cell suspensions were stained for viability using the Zombie Aqua fixable viability kit (1:1000 BioLegend) and fixed with 4% paraformaldehyde (PFA, VWR international) or the Intracellular Fixation and Permeabilization Buffer Set (Thermo Fisher Scientific). Cells were blocked with anti-mouse CD16/32 (93, 1:200, BioLegend) and CD16.2 (9E9, 1:200, BioLegend) followed by staining with a combination of antibodies (see Table S2) for 20 min on ice, washed, and analyzed on an LSR Fortessa cytometer (BD Biosciences) using FlowJo software. For intracellular cytokine staining, cells were cultured for 4 h in 1× cell stimulation cocktail (Thermo Fisher), with protein transport inhibitor cocktail (Thermo Fisher) added for the last 3 h.

### 2.4. Human and mouse adipose-tissue-derived spheroid cultures

Pre-adipocytes isolated from human (3 × 10^4^ cells per well) or murine (5 × 10^4^ cells per well) adipose tissue were seeded into cell-repellent 96-well U-bottom plates (Greiner) to form spheroids over 1-2 days. Spheroids were differentiated into adipocytes using WAT medium with 5 μM dexamethasone, 0.5 μg/mL insulin, 0.5 mM IBMX, and 1 μM rosiglitazone for 2 days, then maintained in WAT medium with 0.5 μg/mL insulin for ∼3 weeks. Three mature spheroids were pooled and stimulated with 5 μg/mL LPS for 2 days for mRNA and cytokine analysis.

### 2.5. Mice

C57BL/6 WT mice were purchased from Charles River. *Wipf1^−/−^* mice were provided by Dr Raif Geha (Boston’s Children Hospital, Boston, USA)^17^ and backcrossed on C576BL/6 mice for at least eight generations. C3H/HeJ mice were purchased from the Jackson Laboratory. All mice were housed and bred at the Specific Opportunist Pathogen Free (SOPF) animal facility at the TranslaTUM. Mice aged 6-16 weeks were used for all experiments. To induce colitis in *RAG1^−/−^* recipients, 5 × 10^5^ sorted naive CD4 T cells (CD19^-^CD4^+^CD25^-^CD45RB^hi^) were injected alone or in combination with MACS purified (purity more than 97%) 3 × 10^5^ WT or *Wipf1^−/−^* B cells. Mice were monitored for weight loss and end analysis was performed 6 weeks post injection. B cells were depleted from *Wipf1^−/−^* mice by i.p. administration of 250 µg/mL anti-CD20 (SA271G2, Biolegend) or rat IgG2b, k Isotype control (RTK4530, Biolegend) at 6 and 8 weeks of age; end analysis was performed at 10 weeks of age.

### 2.6. Murine primary cell isolation and culture

Gonadal and mesenteric WAT (gWAT and mWAT) were harvested from mice, minced, and enzymatically digested in 3 mL digestion medium (HBSS with Ca^2+^/Mg^2+^, 10 mM HEPES, 5% FCS [v/v], 1% penicillin-streptomycin [v/v], 3% BSA [w/v], 1 mg/mL collagenase D, 0.5 mg/mL dispase II, 0.25 mg/mL DNase I) for 40 min at 37 °C with interval shaking. The suspension was filtered through 70- and 40-μm strainers (Greiner), separating tissue fragments and mature adipocytes from the stromal vascular fraction (SVF). Filters were rinsed with 5 mL WAT medium (advanced DMEM/F12, 1% GlutaMAX [v/v], 5% FCS [v/v], 1% penicillin-streptomycin [v/v]), and the SVF was pelleted by centrifugation at 1200 rpm for 10 min. The pellet was resuspended in WAT medium and plated in 10-cm dishes to expand adherent pre-adipocytes, with medium changes every 2-3 days until confluence. For mature adipocyte enrichment, tissue was minced, digested, filtered through a 100-μm strainer, and centrifuged at 50 *g* for 5 min at 4 °C. Floating mature adipocytes were collected with wide-bore pipette tips, washed with WAT medium, and stored at 4 °C. Lymphocytes were isolated from the SVF by Percoll gradient centrifugation (2200 rpm, 20 min, 4 °C, 1 acceleration, 0 deceleration), collected at the 40/80% interface, and washed in B cell medium (RPMI-1640 + GlutaMax, 10 mM HEPES, 10%FCS, 1 × P/S and 0.05 mM 2-mercaptoethanol).

Splenic resting, naive B cells were purified using negative B cell isolation kits yielding enriched populations of 95%-98% (Miltenyi Biotec cat. no.: 130-090-862).

Murine colonic lymphocytes were isolated as follows. Colons were harvested, cleared of fat and luminal contents, opened longitudinally, and cut into ∼0.5-cm pieces. Epithelial cells were removed by incubating the tissue pieces for 30 min at 37 °C with rolling in Ca^2+^/Mg^2+^-free HBSS containing 10 mM HEPES, 5% FCS, 1× penicillin-streptomycin, 5 mM EDTA, and 1 mM DTT. Tissue was then washed by vortexing in Ca^2+^/Mg^2+^-rich HBSS with 10 mM HEPES, 1× P/S, and 5% FCS. Minced tissue was enzymatically digested for 30 min at 37 °C on a thermal block using 0.5 mg/mL collagenase D, 0.5 mg/mL DNase I, and 1 mg/mL Dispase II. The suspension was mechanically dissociated and passed through a 100-µm strainer, and lymphocytes were enriched via Percoll (GE Healthcare) gradient centrifugation (25 min, 2200 rpm, 4 °C), collecting the 40/80% interface and cells washed in B cell medium.

For the generation of bone marrow-derived macrophages (BMDMs), bone marrow was isolated from the hind legs of C3H mice. After removing muscles and connective tissue, bone marrow was flushed from the femur and tibia with PBS through a 70-μm filter. Cells were centrifuged (1200 rpm, 5 min, 4 °C), and cultured in B cell medium with 40 ng/mL murine macrophage colony-stimulating factor (M-CSF) for 7 days to differentiate into M0 macrophages.

All cells were cultured at 37 °C and 5% CO_2_. Pre-adipocytes and mature adipocytes were maintained in WAT medium, while BMDM and B cell experiments used B cell medium. Adherent cells were split with 0.05% EDTA in PBS after reaching confluence. Viable cells were counted using trypan blue and a hemocytometer. For cryopreservation, cells were stored in FCS with 10% DMSO in liquid nitrogen.

### 2.7. B cell/adipocyte co-cultures

A transwell culture system was used for co-culture experiments. Isolated mature adipocytes (20 μL) were pipetted onto inverted Transwells (0.4 μm, Sarstedt) and placed in 24-well plates with 0.5-1 mL B cell medium. After 4 h, 8 × 10^5^ purified B cells were added to each membrane. Co-cultures were stimulated with 5 μg/mL LPS for 4 days.

For neutralization assays, 5 μg/mL neutralizing antibody (anti-IL6, anti-IL10, anti-TNFα, or anti-IFNγ) was added at day 0 and day 2. B cells were collected for FACS analysis, and supernatants were used for cytokine profiling (LEGENDplex multiplex assays), macrophage migration and polarization assays, and lactate concentration measurements (YSI Model 2950 Analyser).

### 2.8. Macrophage assays

Macrophage migration was assessed using 8-μm Transwell inserts (Sarstedt). Supernatants from Transwell co-culture experiments were used as chemoattractants in 24-well plates. Then, 1 × 10^5^ M0 macrophages were added to the upper chamber and incubated for 4 h at 37 °C. Non-migrated cells were removed, and migrated cells on the underside were stained with Hoechst for 10 min and counted at three random sites using a fluorescence microscope (EVOS FL). Cell counts were normalized to the insert surface area (mm^2^).

For macrophage activation/polarization with Transwell co-culture supernatants, 1 × 10^5^ M0 macrophages were cultured with an equal 100-μl co-culture medium volume for 12-18 h at 37 °C. Cells were lysed in RLT buffer for reverse transcription, quantitative polymerase chain reaction (qPCR) analysis.

### 2.9. RNA extraction, qPCR

Total RNA was isolated from 1 × 10^5^ polarized macrophages or pooled adipose spheroids using the RNeasy Micro Kit (Qiagen), with tissue disrupted in RLT buffer. Then, 10 μL RNA was reverse transcribed to cDNA (High-Capacity cDNA Reverse Transcription Kit, Applied Biosystems). qPCR was performed with Takyon No ROX SYBR 2x MasterMix (Eurogentec) and specific primers (Table S3) on a Light Cycler 480II (Roche). Relative gene expression was calculated using the 2^−ΔCt^ method with *Hprt* as housekeeping genes.

### 2.10. Multiplex assay

Cytokines and immunoglobulins in culture supernatants were quantified using LEGENDplex multiplex assays (BioLegend) as per the manufacturer’s instructions. For murine samples, we used the Mouse Inflammation (13-plex) or Immunoglobulin Isotyping (6-plex) panels; for human samples we used the Human Adipokine Panel (13-plex).

### 2.11. Non-targeted metabolomics

Non-targeted metabolomics was performed on extracts from spheroid culture supernatants. Polar metabolites were extracted with ice-cold 80% methanol, vacuum-concentrated, and stored at –70 °C until measurement. For LC-MS analyses an Agilent 1290 Infinity II UHPLC inline with a Bruker Impact II QTOF-MS operating in negative ion mode was used. Scan range was from 20 to 1050 Da. Mass calibration was performed at the beginning of each run. LC separation was on a Waters Atlantis Premier BEH ZHILIC column (100 × 2.1 mm, 1.7-µm particles), buffer A was 20 mM ammonium carbonate and 5 µM medronic acid in milliQ H_2_O and buffer B was 90:10 acetonitrile: buffer A and the solvent gradient was from 95% to 55% buffer B over 14 min. Flow rate was 180 µL/min, column temperature was 35 °C, autosampler temperature was 5 °C, and injection volume was 3 µL. Data processing including detection, deconvolution, and identification of features was carried out using Metaboscape 2023 (Bruker). At least three negative controls were run per experiment.

### 2.12. Single-cell RNA sequencing

For single cell sequencing, CD45+ immune cells from WAT were sorted on the BD Aria. Single cell suspension and library preparation were performed with 20 000 of these cells on the 10× genomics platform and following the Chromium Next GEM Single Cell 3’ Reagent Kit User Guide Revision E. Illumina Next Generation Sequencing was performed using an Illumina NovaSeq 6000 sequencing system by the Helmholtz Munich Core Facility Genomics, including raw data quality control, alignment, and mapping using Cell Ranger. The resulting data were then quality controlled and analyzed using the scanpy toolkit^18^ and decoupleR.^19^

### 2.13. Human MAT single-cell RNA sequencing re-analysis

An annotated gene expression matrix of immune cells^6^ was loaded, processed, and re-analyzed using the scanpy toolkit (ver. 1.11.2,^20^ SCR_01813999), scvi-tools (ver. 1.3.1, SCR_026673), and other common Python (ver. 3.13, SCR_008394) toolboxes.

Low-quality observations, filtered individually per sample based on observed distribution by number of expressed unique genes (minimum: 500-1250, maximum: 3500-7500), total read counts (maximum: 11 500-50 000), and percentage of mitochondrial genes (maximum.: 15%-18%) and genes expressed by fewer than three cells were removed. For sample integration highly variable genes (flavor: “seurat_v3,” n_top_genes: 15 000, layer: “counts,” batch_key: “sample_ID,” subset: True) were calculated and a single cell Variational Inference (scVI^21^) model (layer: “counts,” batch_key: “sample_ID,” n_latent: 30, n_layers: 2) was trained (max_epochs: 400, early_stopping: True, early_stopping_patience: 20, early_stopping_monitor: “elbo_validation”). Next, the neighborhood graph (nearest neighbors: 50) based on the scVI model’s latent space representation, Uniform Manifold Approximation and Projection (UMAP; SCR_018217103; min. distance: 0.4, neg. edge sample rate: 12), and initial Leiden clustering (ver. 0.10.2; resolution: 1.0) were computed as indicated and used as the basis for cell type annotations in combination with computed marker genes (method: “wilcoxon,” corr_method: “bonferroni”). For displays of gene expression, normalized values were extracted from the integration model (library_size: 1e4), log1p transformed, and scaled (max_value: 10, zero_center: False).

### 2.14. Statistics

GraphPad Prism 11.0. was used to generate graphs and for all statistical analyses other than those performed on the RNA sequencing data. Figure legends detail the number of experimental replicates, *n* values, and statistical test used. The choice of statistical test was based on the experimental design, distributional characteristics of the data, and assay detection limits. Model assumptions were assessed by inspection of Q-Q and residual plots. For comparisons between two matched conditions, data were analyzed using a paired two-tailed t-test when the paired differences were reasonably compatible with a normal distribution; two independent groups were analyzed using an unpaired t-test with Welch’s correction. For three or more matched conditions, a one-way repeated-measures ANOVA with Geisser–Greenhouse correction was used, followed by prespecified multiple comparisons using the Holm–Šídák method. If the distribution of the data was nonparametric after testing, two matched conditions were compared using the Wilcoxon matched-pairs signed-rank test, whereas two independent groups were compared using the Mann–Whitney test. For three or more matched conditions, the Friedman test followed by Dunn’s multiple-comparisons test was used. Cytokine concentrations within the quantifiable range were log_10_-transformed prior to parametric analysis. For cytokines containing measurements below the assay limit of detection (LOD), rank-based nonparametric analyses were used on untransformed data. For transparency and biological interpretability, the figures continue to display the original cytokine concentrations in pg/mL. Where multiple cytokines were tested for the same biological comparison, *P* values were adjusted for multiple testing using the Holm–Šídák method. All statistical tests were two-sided, and adjusted *P* < .05 was considered statistically significant. Error bars represent mean ± SD unless otherwise indicated in the figure legends: *P < .05, **P < .01, ***P < .001, ****P < .0001.

## 3. Results

### 3.1. B cells accumulate in human CrF

Adipose tissue is composed of mature adipocytes and the SVF. The SVF contains immune cells and a heterogeneous population of mesenchymal stem cells, including committed pre-adipocytes that can differentiate into adipocytes.^22^ To characterize the composition of immune cells in adipose tissue in CD patients on a cellular level, we analyzed biopsies from CrF around inflamed and stenotic intestinal segments and compared them to uninvolved MAT from the resection margin from the same patient (Figure 1A, patients’ characteristics summarized in Table 1 and Table S1). Using multi-color flow-cytometry, we observed a significantly increased number of CD45-expressing leukocytes in CrF tissue compared to MAT (Figure S1A + B), with an overabundant population of CD19^+^/HLA-DR^+^ B cells (Figure 1B + C), IgA^+^/CD19^low^ and IgA^+^/CD19^high^ class-switched B cells (Figure 1D + E, Figure S1C). Our analysis also revealed variable amounts of CD3^+^ T cells (Figure 1F; Figure S1D), and CD206^+^/HLA-DR^+^ macrophages (Figure 1G + H) in CrF relative to MAT. Different treatment regimens (including the biologics anti-TNF-a [infliximab or adalimumab] or anti-IL-12/23 [ustekinumab]) or glucocorticoids did not seem to influence populations of cells isolated (color coded in Figure 1C–F, H). Thus, we found accumulation of different immune cells, especially B cells, in CrF compared to MAT in CD patients.

**Figure 1.**
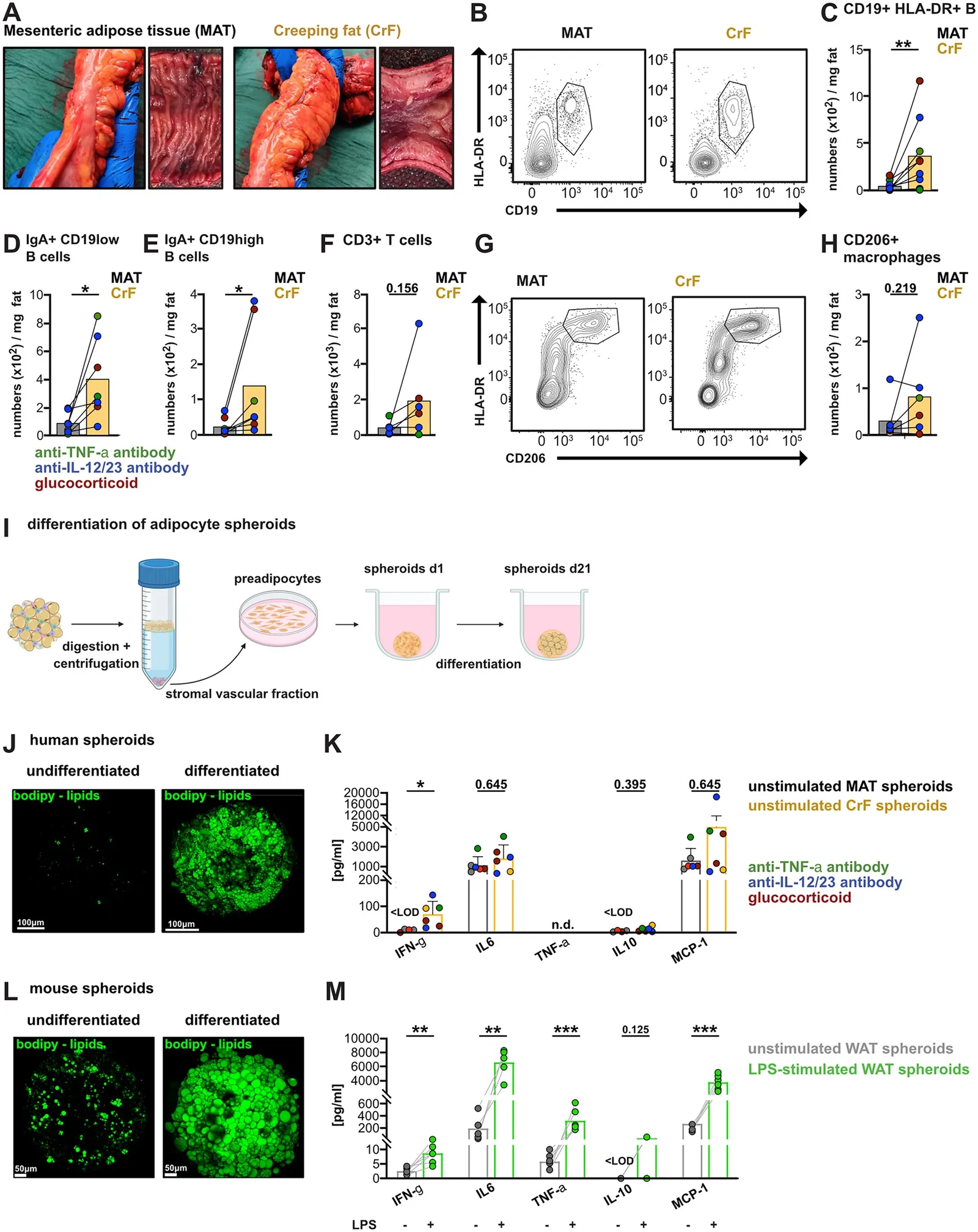
Immune cell infiltration and adipocyte inflammation in creeping fat of CD patients. (A) Representative images of MAT of a CD patient. Left side: uninvolved intestinal segment (MAT, external and mucosal perspectives). Right side: macroscopic characteristics of a small bowel segment with attached CrF and a stricture visualized from the mucosal aspect. (B) Representative flow plots of human CD45^+^/CD11b^-^/CD19^+^/HLA-DR^+^ B cells, (C) quantified as cell counts per mg fat isolated from MAT and CrF. (D + E + F) Total cell counts of (D) CD45^+^/CD11b^-^/IgA^+^/CD19^-^, (E) CD45^+^/CD11b^-^/IgA^+^/CD19^+^ class-switched B cells and (F) CD45^+^/CD11b^-^/CD3^+^ T cells per mg fat isolated from MAT and CrF. (G + H) Representative plots of CD45^+^/CD11b^+^/CD206^+^/HLA-DR^+^ macrophages, (H) quantified as cell counts per mg fat. Paired samples from MAT and CrF were collected from each patient (*n* ≥ 7); bars indicate the mean. Treatment indicated by color coding. Statistical significance was calculated using Wilcoxon signed-rank test. (I) Experimental setup for adipose tissue-derived spheroid cultures. (J) Representative immunofluorescence images of human spheroids at day 1 (undifferentiated spheroids, left) and day 21 (differentiated spheroids, right) stained with BODIPY (green) for lipid visualization. (K) Cytokine secretion profile in the supernatants of differentiated MAT- or CrF-derived adipocyte spheroids was measured using a multiplex assay (mean ± SD). For comparison purposes, cytokines of supernatants of unstimulated MAT-derived spheroids plotted are the same in K and Figure S1F. Spheroids were generated from *n* ≥ 5 patients, with two distinct tissue groups (MAT, CrF) per patient. Treatment indicated by color coding. Cytokines satisfying the assumptions for parametric analysis were analyzed using a unpaired t-test with Welch’s correction on log_10_-transformed concentrations; cytokines for which these assumptions were not met (values < limit of detection [LOD]) were analyzed using a Mann–Whitney test. Multiple testing was corrected for by Holm–Šídák across all *P*-values in the graph. Statistical tests were two-sided. (L) Representative immunofluorescence images of murine adipocyte spheroids from WT gWAT at day 1 (undifferentiated spheroids, left) and day 21 (differentiated spheroids, right) stained with BODIPY (green) for lipid visualization. (M) Cytokine secretion profile in the supernatants of differentiated unstimulated or LPS-stimulated gWAT-derived adipocyte spheroids was measured using a multiplex assay (mean). Spheroids were generated from *n* ≥ 5 WT mice, pooled from three independent experiments. Cytokines satisfying the assumptions for parametric analysis were analyzed using a paired t-test on log_10_-transformed concentrations; cytokines for which these assumptions were not met (values < LOD) were analyzed using the Wilcoxon matched-pairs signed-rank test. Multiple testing was corrected for by Holm–Šídák across all *P*-values in the graph. Statistical tests were two-sided. *P < .05, **P < .01, ***P < .001, ****P < .0001. Illustration I was created with BioRender.com.

### 3.2. Human adipose tissue pathology imprints MAT-derived spheroids to secrete pro-inflammatory cytokines *in vitro*

To investigate human adipose tissue inflammation in more detail *in vitro*, we used a pre-adipocyte spheroid culture system. For this, we isolated the SVF of human MAT or CrF, expanded the SVF into pre-adipocytes, and cultured them as self-organizing 3D spheroids followed by differentiation into adipocytes (Figure 1I–K). Lipid uptake was verified using BODIPY staining (Figure 1J). Secreted cytokines and adipokines in the culture supernatant were measured using a multiplex assay. We detected a significantly higher concentration of the pro-inflammatory cytokine IFN-γ and the adipokines leptin and adiponectin in the culture supernatants of CrF-derived adipocyte spheroids compared to MAT-derived adipocyte spheroids. Interestingly, the highest concentrations of cytokines measured in the culture supernatant of both, MAT and CrF-derived adipocyte spheroids, were of IL-6 and of the monocyte/macrophage chemoattractant protein (MCP-1), whereas levels of TNF-α and IL-10 were very low (Figure 1K, Figure S1E). Treatment regiments of the patients varied and did not seem to have an effect on cytokine secretion (color coded in Figure 1K, Figure S1E). We concluded that adipocyte spheroids from CrF of CD patients readily secreted enhanced levels of IFN-γ, adiponectin, and leptin, despite prolonged *in vitro* culture conditions.

The formation of CrF has been suggested to be a response mechanism against bacterial translocation from a leaky intestinal barrier.^1^^,^^23^ Consequently, to mimic bacterial infiltration, we next stimulated human MAT-derived adipocyte spheroids with LPS, a bacterial cell wall component. We detected about a 10× higher concentration of IL-6 (from 1000 to 10 000 pg/mL), MCP-1 (from 1500 to 15 000 pg/mL), and enhanced concentrations of IFN-γ, adiponectin, and leptin, but very little TNF-α or IL-10 in the culture supernatants of LPS-stimulated compared to unstimulated human MAT-derived spheroids (Figure S1F + G). Consequently, LPS stimulation of MAT-derived adipocyte spheroids mimicked the cytokine secretion profile of unstimulated CrF-derived adipocyte spheroids (compare Figure S1F to 1K).

Due to the limited availability of human samples and to model human WAT inflammation *in vitro*, we went on to investigate murine adipocyte inflammation. First, we isolated mature adipocytes from WT mice, cultured them in a transwell culture system and stimulated them with LPS (Figure S1H-J). We found significantly increased levels of IL-6, TNF-α, and MCP-1 secreted into the supernatant of mature adipocyte cultures in the presence of LPS (Figure S1J). In parallel, we isolated pre-adipocytes from WAT of WT mice (Figure 1I), differentiated them into 3D spheroids, and subsequently stimulated them with LPS (Figure 1L + M). Similar to human adipocyte spheroids, LPS-stimulation of mouse WAT-derived adipocyte spheroids led to a more than 10× higher concentration of IL-6 (from 200 to 6000 pg/mL) and MCP-1 (from 200 to 4000 pg/mL), and significantly enhanced concentrations of IFN-γ and TNF-α, but very little IL-10 in the culture supernatants of LPS-stimulated compared to unstimulated mouse adipocyte spheroids (Figure 1M). Of note, the cytokine secretion profile of mouse adipocyte spheroids after LPS stimulation (Figure 1M) was very similar to what we observed after LPS stimulation of human MAT-derived adipocyte spheroid cultures (Figure S1F), or even unstimulated CrF-derived adipocyte spheroids (Figure 1K). Thus, *in vitro* LPS stimulation of MAT-derived murine or human adipocyte spheroids mimicked the cytokine secretion profile of human unstimulated CrF-derived adipocyte spheroids. Together, we here describe novel 3D *in vitro* culture systems to study human, or mouse, adipocyte inflammation.

### 3.3. B cells contribute to M2-like macrophage accumulation in gut-associated WAT in mouse models of intestinal inflammation

We have previously described mice lacking the Wiskott–Aldrich syndrome protein interacting protein (*Wipf1^−/−^* mice), a genetically engineered mouse that develops intestinal inflammation, as model for human IBD.^24^ Intestinal inflammation observed in *Wipf1^−/−^* mice is characterized by immune cell infiltration (including neutrophils and mast cells) within the mucosa and submucosa of the colon, thickening of the muscularis interna, as well as irregular crypt architecture featuring increased crypt length, crypt abscesses, and decreased goblet cell numbers, resulting in a mild-to-moderate colitis severity score (Figure S2A–F—adapted from the literature^24^). Furthermore, we observed large B cell-containing isolated lymphoid follicles, also comprising T cells, in the colon of *Wipf1^−/−^* mice (Figure S2G—adapted from the literature^24^). In addition to increased rigidity of the inflamed intestinal tissue, formation of lymphatic structures in the mesenteric WAT (mWAT) of *Wipf1^−/−^* mice was occasionally noted (example shown in Figure S3B–C). Furthermore, we detected an increased relative mRNA expression of the pro-inflammatory cytokines IFN-γ and TNF-α in isolated WAT of *Wipf1^−/−^* mice (Figure S3D), all pointing at IBD-associated WAT inflammation.

To characterize the composition of immune cells in adipose tissue of *Wipf1^−/−^* mice, we isolated the SVF from WAT. Similar to our findings in human IBD-associated MAT biopsies (Figure 1A–H), we found a significant increase in B cells but no significant differences in CD4 T cells (Figure 2A–D, Figure S3E) in the SVF isolated from mesenteric WAT of *Wipf1^−/−^* mice compared to homeostatic tissue using flow cytometry. Concomitantly, we found a substantial population of CD206-expressing, M2-like macrophages (Figure 2E + F) in inflamed WAT compared to controls, which we confirmed using tissue clearing and volumetric imaging (Figure 2G + H).^25^ The infiltration of immune cells, especially B cells and M2-like macrophages, as well as the enhanced pro-inflammatory cytokine secretion in IBD-associated WAT of *Wipf1^−/−^* mice, similar to what we observed in CrF of CD patients, indicated a prominent adipose tissue inflammation in the *Wipf1^−/−^* mouse model.

**Figure 2.**
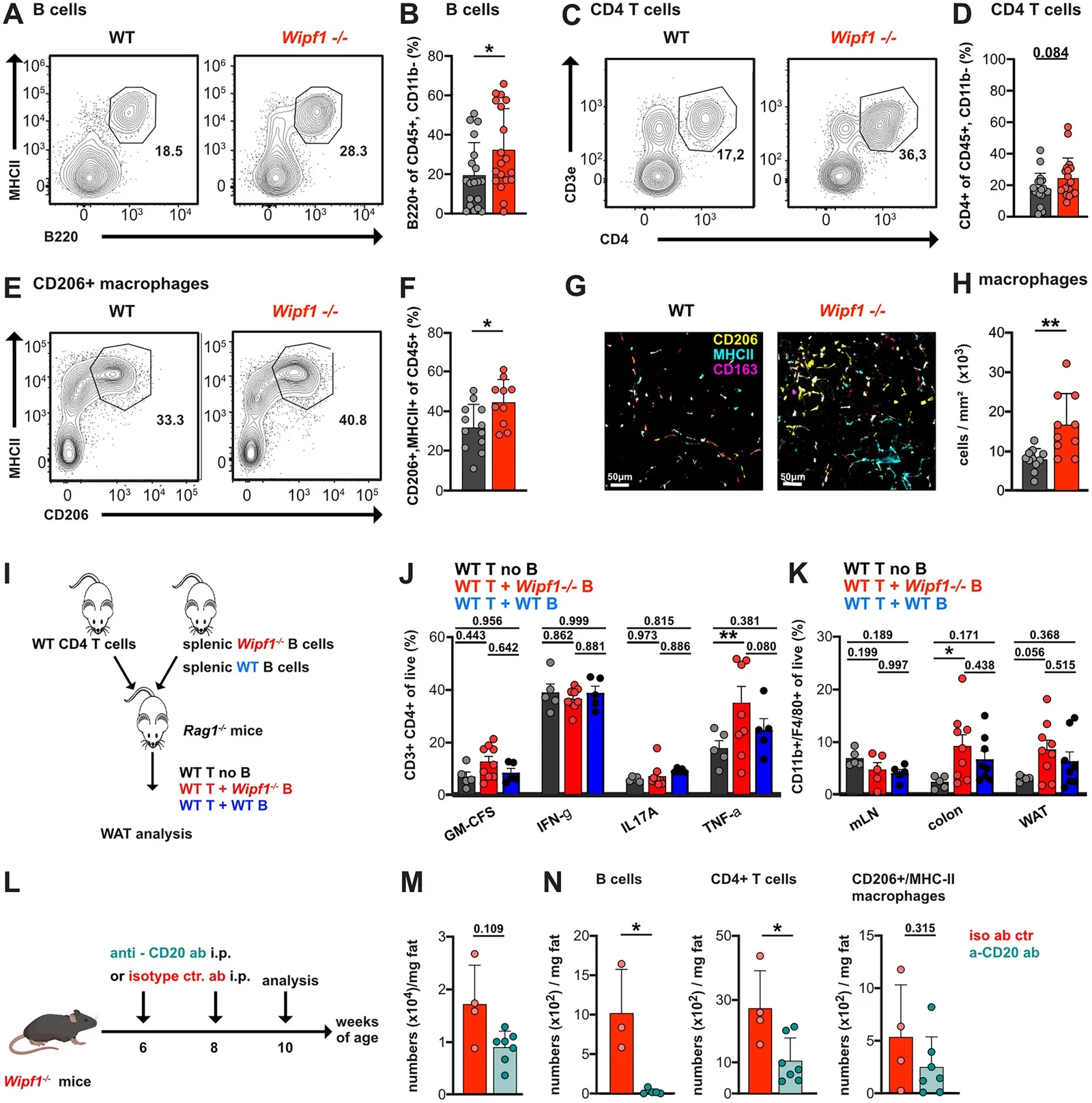
B cells contribute to M2-like macrophage accumulation in gut-associated WAT in mouse models of intestinal inflammation. (A) Representative flow plots of CD45^+^, CD11b^-^, B220^+^, and MHC-II^+^ B cells isolated from mWAT from WT and *Wipf1^−/−^* mice quantified in B (frequencies, mean ± SD). (C) Representative flow plots of CD45^+^, CD11b^-^, CD3e^+^, and CD4^+^ T cells isolated from mWAT from WT and *Wipf1^−/−^* mice quantified in D (frequencies, mean ± SD). (E) Representative flow plots of CD45^+^, CD11b^+^, CD206^+^, and MHC-II^+^ M2 macrophages quantified in F (frequencies, mean ± SD) (A–F), *n* ≥ 10 mice per genotype, pooled from multiple independent experiments. (G) Confocal microscopy of cleared gWAT of WT and *Wipf1^−/−^* mice showing CD206^+^ (yellow), CD163^+^ (magenta), and MHC-II^+^ (cyan) monocytes/macrophages quantified in H (mean ± SD); *n* = 6 mice/genotype; 1-2 3D images per mouse. (I) Experimental setup of the adoptive transfer of naive T cells together with splenic WT B cells, *Wipf1^−/−^* B cells, or without B cells into *Rag1^−/−^* recipients to induce colitis. (J) Frequencies of CD3^+^,CD4^+^ T cells isolated from mWAT expressing indicated cytokines intracellularly after *in vitro* re-stimulation. (K) Frequencies of CD11b^+^, F4/80^+^ monocytes/macrophages isolated from mLNs, colonic tissue or mWAT; *n* ≥ 5 per genotype, pooled from two independent experiments. (L) Experimental design of B cell depletion in *Wipf1^−/−^* mice using anti-CD20 or isotype control antibodies. (M) Total cell numbers/mg fat tissue isolated from mWAT. (N) Total number/mg mWAT of CD45^+^, CD11b^−^, CD19^+^, MHC-II^+^ B cells, CD45^+^, CD11b^-^, CD3^+^, CD4^+^ T cells and CD45^+^, CD11b^+^, CD206^+^, MHC-II^+^ macrophages isolated from mWAT of treated *Wipf1^−/−^* and control mice; *n* ≥ 3 per group, pooled from three independent experiments. Statistical significance was calculated using (B, D, F, H) unpaired Student’s t test, (J, K) ordinary two-way ANOVA with Tukey’s multiple comparison test, or (M-N) Mann–Whitney test. *P < .05, **P < .01, ***P < .001, ****P < .0001. Illustration L was created with BioRender.com.

To address the influence of B cells on immune cell recruitment into adipose tissue in an acute model of colitis, we made use of the T cell transfer colitis model. In this model, the transfer of naive CD4^+^CD45RB^high^ WT T cells in the absence of regulatory T cells generates acute intestinal inflammation.^20^ We co-transferred purified B cells isolated from the spleen of WT or *Wipf1^−/−^* mice together with naive WT T cells into *Rag1^−/−^* recipients and analyzed cell populations isolated from mesenteric WAT, mesenteric lymph nodes (mLN), or colon 6 weeks after transfer (Figure 2I). Weekly scoring of the mice demonstrated an initial weight gain followed by a drop in weight of *Rag1^−/−^* recipients co-transferred with WT B cells, whereas mice co-transfered with *Wipf1^−/−^* B cells remained at a stable weight and recipients of no B cell co-transfer lost some weight (Figure S3G). *Rag1^−/−^* recipients demonstrated comparable colon weight/length ratios, independent of the co-transfer of B cells (Figure S3H). In line with our previously published findings,^24^ B cell co-transfer in the *Rag1^−/−^* T cell colitis model resulted in the differentiation of B cells into antibody-secreting cells, which we detected in cells isolated from the mLN, colonic lamina propria, and mWAT using flow cytometry (Figure S3I). Frequencies of CD4 T cells were similar between the three organs analyzed (Figure S3J). After *in vitro* restimulation of cells isolated from mWAT or mLN, but not colon, of *Wipf1^−/−^* B cell co-transferred mice, CD4 T cells displayed enhanced frequencies of pro-inflammatory cytokine-expressing cells, particularly TNF-α but not IFN-γ or IL-17A (Figure 2J, Figure S3K-M), indicating enhanced activation of CD4 T cells in the presence of B cells. Interestingly, in WT but especially in *Wipf1^−/−^* B cell co-transferred recipients, frequencies of CD11b^+^/F4/80^+^ macrophages (Figure 2K), but not other CD11b^high^ myeloid cells (Figure S3N) were noticeably increased in cells isolated from the colon and mWAT, but not mLN (Figure 2K). From these findings, we concluded that the presence of B cells promoted CD4 T cell activation and the accumulation of CD11b^+^/F4/80^+^ macrophages in mWAT in an acute model of colitis.

Given the enrichment of B cells in human CrF and IBD-associated mouse WAT, we next assessed whether B cells contribute to adipose tissue inflammation in the *Wipf1*^−/−^ mouse model. B cells were depleted at 6 and 8 weeks of age using an anti-CD20 antibody or an isotype control antibody (Figure 2L). In mWAT of anti-CD20-treated *Wipf1*^−/−^ mice, B cell numbers were efficiently reduced, accompanied by lower total CD45^+^ leukocyte counts (Figure 2M + N). WAT weight was unchanged between anti-CD20 and isotype-treated groups (Figure S2O). Similar to our previous observations in the colonic lamina propria,^24^ B cell depletion significantly reduced CD4 T cell numbers in mWAT (Figure 2N, Figure S3P), but the reduction in CD206^+^ macrophage numbers and frequencies did not reach statistical significance (Figure 2N, Figure S3P). These data indicated that B cell depletion is associated with reduced CD4 T cell accumulation in IBD-associated WAT.

Together, results from *in vivo* experiments demonstrated that the presence of B cells affects the accumulation of CD4 T cells as well as macrophages in IBD-associated WAT.

### 3.4. B cell/adipocyte crosstalk promotes B cell activation and adipocyte cytokine secretion

Our *in vivo* data suggested that the presence of B cells in adipose tissue might drive the accumulation of CD4 T cells and macrophages during inflammation. We next used our *in vitro* adipocyte culture systems to investigate the B cell/adipocyte crosstalk. For this, we co-cultured naive splenic WT or *Wipf1^−/−^* B cells together with primary mature adipocytes isolated from gonadal, non-inflamed WAT (gWAT) of WT mice for 4 days using a transwell assay and stimulated B cells and adipocytes using LPS (Figure S4A).

To determine how B cells affect adipocyte activation, we measured cytokine concentrations in supernatants from LPS-stimulated adipocytes cultured alone or together with WT or *Wipf1^−/−^* B cells (Figure S4B + C). LPS-stimulated adipocytes alone produced little IL-10 or TNF-α and both cytokines were increased only when B cells were stimulated with LPS. IL-6 was induced by LPS stimulation in adipocytes and the presence of B cells further increased IL-6 concentrations in the culture supernatant (Figure S4B + C). In contrast, MCP-1 was detected in supernatants from LPS-stimulated adipocytes, but not from B cells cultured alone (Figure 3A + B). Interestingly, MCP-1 concentrations increased significantly when adipocytes were co-cultured with WT or *Wipf1^−/−^* B cells in the presence of LPS (Figure 3A + B). Together, these data indicate that activated B cells enhance MCP-1 secretion by adipocytes through soluble, contact-independent signals.

**Figure 3.**
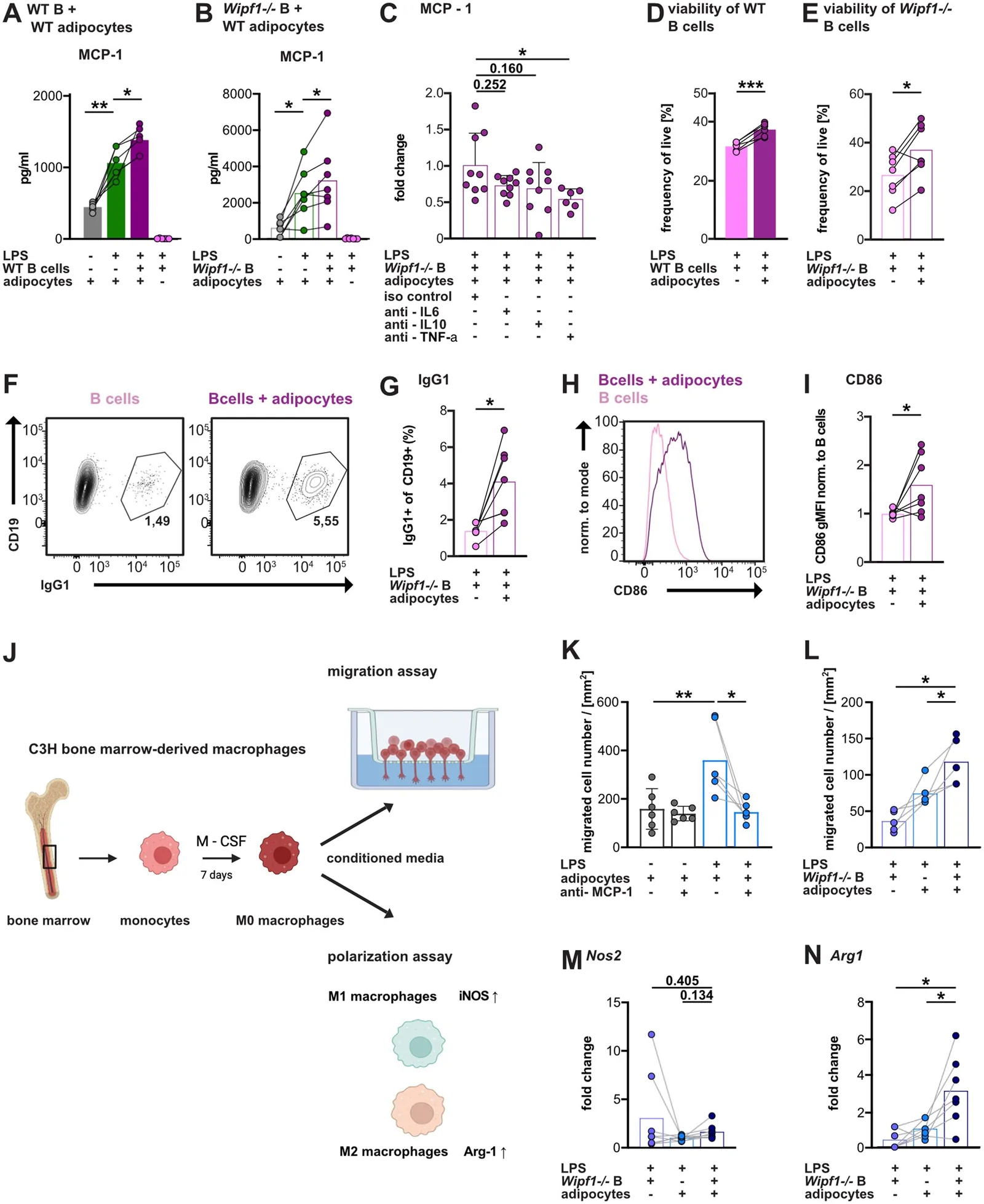
B cell/adipocyte crosstalk promotes B cell activation and adipocyte cytokine secretion, resulting in M2-like macrophage polarization. (A) MCP-1 concentrations in the culture supernatants of WT B cells co-cultured with WT adipocytes. *n* = 6 mice pooled from two independent experiments. (B) MCP-1 concentrations in the culture supernatants of *Wipf1^−/−^* B cells co-cultured with WT adipocytes. *n* ≥ 7 mice pooled from four independent experiments. Statistical significance was calculated using (A, B) one-way repeated measures ANOVA with Geisser–Greenhouse correction, followed by Holm–Šídák correction. The B cell + LPS condition is included in the graph for descriptive comparison but was excluded from statistical testing as it is not part of the matched adipocyte comparison. (C) MCP-1 concentrations in the supernatant of co-cultures of *Wipf1^−/−^* B cells with WT adipocytes in the presence of specified cytokine blocking antibodies normalized to concentrations in the presence of the isotype control *n* ≥ 6 mice pooled from three independent experiments. (D + E) Viability of (D) WT or (E) *Wipf1^−/−^* B cells in co-cultures at day 4 measured by flow cytometry. (F–I) Flow cytometric analysis of *Wipf1^−/−^* B cells cultured with or without adipocytes in the presence of LPS. (F + G) Frequencies of surface IgG1 expression. (H + I) CD86 expression on B cells (I) quantified as geometric mean fluorescence intensity (gMFI) normalized to expression on stimulated B cells. Statistical significance was calculated using (C) ordinary one-way ANOVA with Tukey’s multiple comparison test or (D, E, G, I) paired Student’s t-test. *P < .05, **P < .01, ***P < .001, ****P < .0001. (J) Experimental design of BMDM cultures, migration, and polarization assays. (K + L) Quantification of macrophage migration (K) in the presence of anti-MCP-1 blocking antibody and (L) conditioned media from stimulated *Wipf1^−/−^* B cells, adipocytes, and B cell/adipocyte co-cultures. Cells were counted in three random regions of interest per sample. Data represent the mean from five mice per condition. (M + N) Relative mRNA expression levels (mean fold change) of (M) iNOS (*Nos2*) and (N) Arginase-1 (*Arg1*) 24 h after exposure to conditioned media as indicated, normalized to HPRT and mRNA expression levels after exposure to conditioned media from LPS-stimulated adipocytes. *n* ≥ 7 mice per condition, pooled from four independent experiments. Statistical significance was calculated using (K–N) one-way repeated measures ANOVA with Geisser–Greenhouse correction, followed by Holm–Šídák correction. *P < .05, **P < .01, ***P < .001, ****P < .0001. Illustration J was created with BioRender.com.

To determine whether specific cytokines might influence MCP-1 secretion by adipocytes, we used monoclonal antibodies to selectively block IL-6, IL-10, or TNF-α in co-culture experiments of WT adipocytes and *Wipf1^−/−^* B cells and compared MCP-1 secretion in each condition to that observed in co-cultures treated with an isotype control antibody. We found that only anti-TNF-α antibodies significantly reduced MCP-1 secretion by adipocytes by half when co-cultured with B cells (Figure 3C).

Analyzing the effect of adipocytes on B cells, we found that co-culturing B cells with adipocytes led to an enhanced survival of WT and *Wipf1^−/−^* B cells at day 4 of the co-culture (Figure 3D + E). In addition, *Wipf1^−/−^* B cells exhibited an improved differentiation into class-switched IgG1^+^ B cells (Figure 3F + G), secreted more antibodies into the culture supernatant (Figure S4D), and increased surface expression of the activation marker and co-stimulatory molecule CD86 (Figure 3H + I) when co-cultured with adipocytes, compared to LPS-stimulated B cells alone.

Together, activated B cells in the presence of inflamed adipocytes demonstrate enhanced survival and differentiation. In turn, LPS-stimulated, activated B cells modulated the cytokine secretion profile of adipocytes, leading to increased secretion of MCP-1.

### 3.5. B cell/adipocyte crosstalk promotes migration and polarization of M2-like macrophages

The chemoattractant MCP-1 is involved in the recruitment of macrophages during inflammation. Given that LPS-stimulated adipocytes secrete MCP-1 into the culture supernatant (Figure 1M, Figure S1J), and B cells even enhance this secretion (Figure 3A + B), we next investigated the effect of adipocyte-released mediators on *in vitro* macrophage migration and polarization. For this, we generated BMDMs *in vitro* (Figure 3J). As macrophages themselves are highly sensitive to LPS stimulation, we used bone marrow isolated from C3H/HeJ mice, which do not respond to LPS.^26-28^ Consequently, macrophage polarization and activation was not influenced by LPS but by the soluble factors in the cell culture supernatants of LPS-stimulated adipocytes or B cell/adipocyte cocultures.

Using a transwell assay to study macrophage migration (Figure 3J), we observed 2-fold higher numbers of migrated macrophages/mm^2^ in the presence of conditioned medium from LPS-activated adipocytes compared to unstimulated controls (Figure 3K) after 4 h of culture. Adding anti-MCP-1 blocking antibodies to these cultures abrogated BMDM migration to baseline numbers (Figure 3K). Comparing conditioned medium from LPS-stimulated *Wipf1^−/−^* B cells, adipocytes, or the *Wipf1^−/−^* B cell/adipocyte co-culture, we found that macrophage migration was further enhanced if cultured in conditioned medium of B cell/adipocyte co-cultures (Figure 3L), correlating with the highest concentration of MCP-1 observed (Figure 3B). Together, these results suggest that macrophage migration is mediated by MCP-1 secretion by LPS-simulated adipocytes, which is further enhanced in the presence of B cells.

As we have observed a high prevalence of M2-like macrophages in adipose tissue of *Wipf1^−/−^* mice, we went on to test the influence of adipocyte conditioned media on macrophage polarization (Figure 3J). We found that M0 macrophages cultured for 24 h in the presence of conditioned medium from LPS-activated adipocytes demonstrated higher relative mRNA expression levels of *Nos2* (a marker for M1-like macrophage polarization, Figure S4E) as well as *Arg1* (a marker for M2-like macrophage polarization, Figure S4F) compared to those incubated with conditioned medium from unstimulated adipocytes. Comparing conditioned medium from LPS stimulated *Wipf1^−/−^* B cells, adipocytes, or the *Wipf1^−/−^* B cell/adipocyte co-culture, we found that relative mRNA expression levels of *Nos2* were comparable between all three conditioned media (Figure 3M). Interestingly, mRNA expression levels of *Arg1* were significantly elevated in macrophages exposed to conditioned media of the LPS-stimulated *Wipf1^−/−^* B cell/adipocyte co-cultures, relative to macrophages cultured in conditioned media of LPS-stimulated B cells or adipocytes alone (Figure 3N). These results indicate an enhanced polarization of M2-like macrophages in an environment primed by the B cell/adipocyte crosstalk.

### 3.6. B cell/adipocyte crosstalk contributes to a lactate-rich, inflammatory microenvironment

To investigate which soluble mediators released by inflamed adipocytes could influence the polarization of M2-like macrophages, we went on to analyze the culture supernatants of gWAT-derived spheroids in the presence or absence of LPS using mass spectrometry. After LPS stimulation, we found an increase in orotidine and lactate concomitant with a reduction of citrate in the culture supernatant of adipocyte spheroids (Figure 4A–D). Lactate, a key product of glycolysis, is produced when pyruvate is converted to lactate by the lactate dehydrogenase enzyme (LDHA). Enhanced levels of lactate concomitant with reduced levels of citrate pointed at anerobic glycolysis in hypoxic conditions. Analyzing mRNA expression levels in adipocyte spheroids in the presence or absence of LPS indeed revealed a significant upregulation of the hypoxia marker hypoxia-inducible factor 1 alpha (*Hif1a*) as well as NADPH oxidase 2 (*Cybb*), related to oxidative stress (Figure 4E + F). Together, these findings indicate a hypoxic/inflamed state of LPS-stimulated adipocytes *in vitro*.

**Figure 4.**
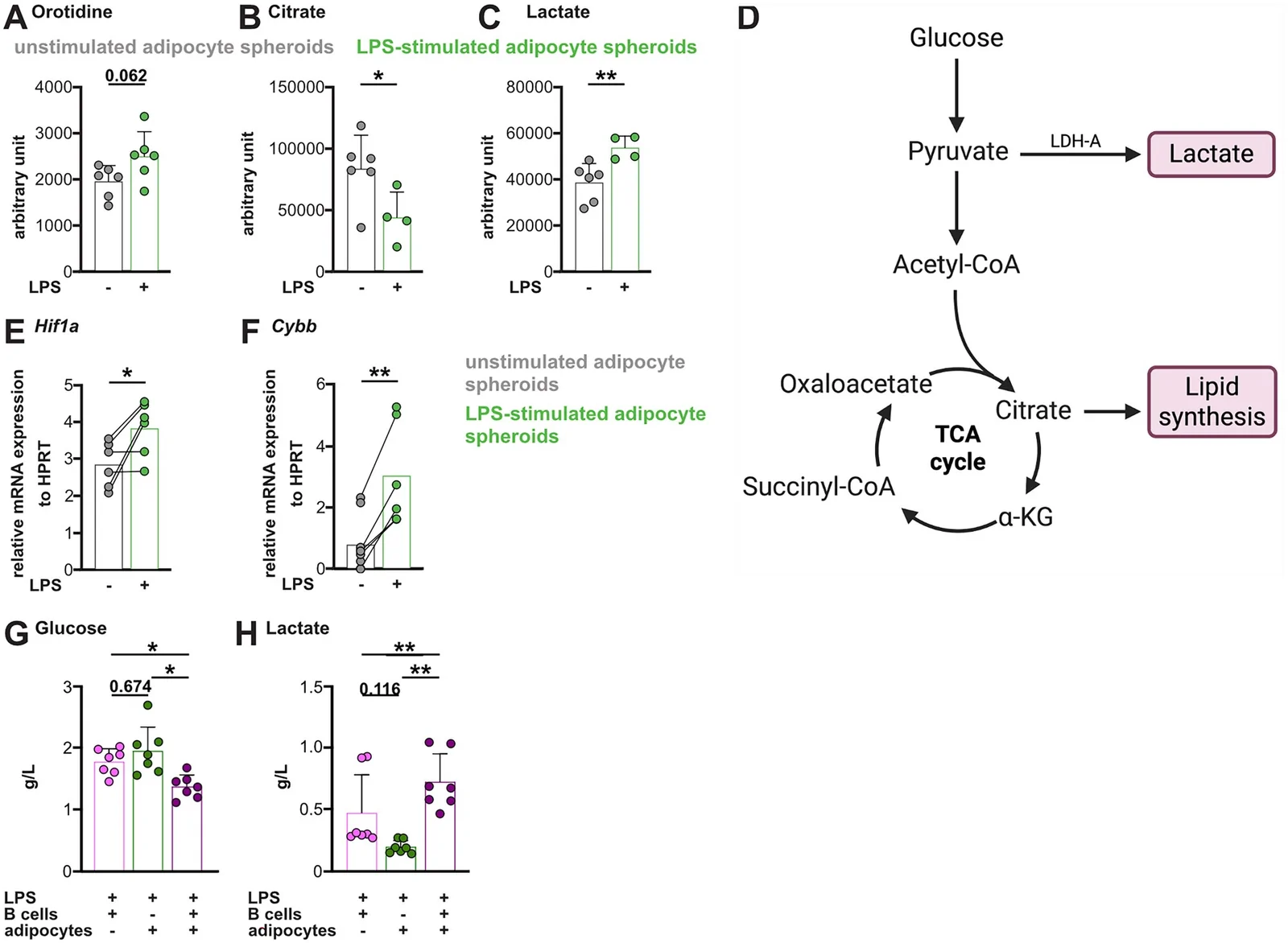
B cell/adipocyte crosstalk contributes to a lactate-rich, inflammatory microenvironment. (A–C) Non-targeted metabolomic analysis of culture supernatants from unstimulated and LPS-stimulated gWAT spheroids. Relative quantification (arbitrary unit ± SD) of (A) orotidine, (B) citrate and (C) lactate levels. Statistical significance was calculated using unpaired Welch’s t-test. (D) Schematic overview of glucose metabolism and TCA cycle. Pyruvate is either converted to lactate via LDH-A or enters the TCA cycle through acetyl-CoA. (E + F) Relative mRNA expression (mean) of (E) *Hif1a* and (F) *Cybb* of non-stimulated and LPS stimulated WAT-derived spheroids, measured by qPCR. Spheroids were generated from *n* ≥ 4 WT mice, pooled from three independent experiments. Statistical significance was calculated using paired Student’s t test. (G + H) Glucose and lactate concentrations were measured in supernatants of LPS-stimulated B cells, adipocytes, or B cell/adipocyte co-cultures using the YSI analyser (g/L ± SD). *n* ≥ 7 mice per condition, pooled from four independent experiments. Statistical significance was calculated by one-way ANOVA with Tukey’s multiple comparison test. *P < .05, **P < .01, ***P < .001, ****P < .0001. Illustration D was created with BioRender.com.

Lactate has been shown to promote the differentiation of M2-like macrophages.^29^^,^^30^ Having observed enhanced mRNA expression levels of Arginase-1 in our BMDM polarization assays in the presence of B cell/adipocyte conditioned medium (Figure 3N), we next determined levels of lactate in the supernatants of the *Wipf1^−/−^* B cell/adipocyte co-cultures. Concomitant with reduced levels of glucose (Figure 4G), we found an increased concentration of lactate in the B cell/adipocyte conditioned medium compared to medium of B cells or adipocytes alone (Figure 4H), which might enhance M2-like macrophage polarization.

### 3.7. scRNAseq reveals increased LDHA and HIF1A mRNA in mouse IBD WAT and human CrF and a possible CCL2/CCR2-driven macrophage recruitment

Results from our *in vitro* analysis suggested that B cell/adipocyte crosstalk enhances the activation of both, B cells and adipocytes, leading to increased amounts of MCP-1 and lactate. This created a lactate-rich, inflammatory microenvironment that in turn supported the migration and polarization of M2-like macrophages *in vitro*. To assess whether this phenotype and the underlying mechanisms are also present in *ex vivo* IBD-associated WAT from both mice and humans, we next performed single-cell RNA sequencing (scRNAseq) analysis. Uniform manifold approximation and projection (UMAP) analyses confirmed the presence of all major immune cell subsets in the gWAT of both WT and *Wipf1^−/−^* mice (Figure 5A, Figure S5A–C). Differential gene expression (DGE) analysis, focusing on hallmark gene sets within the B cell cluster, revealed a significant enrichment of transcripts associated with inflammation and pro-inflammatory cytokine responses. Notably, pathways related to IL-2/STAT5, TNF-α, and IFN-γ signaling were upregulated in B cells from inflamed gWAT of *Wipf1^−/−^* mice compared to WT controls (Figure 5B). Further pathway activity inference within the B cell cluster demonstrated a pronounced hypoxia signature, along with upregulation of various cytokine signaling pathways, including JAK/STAT, NF-κB, PI3K, and TGF-β, in B cells from *Wipf1^−/−^* WAT (Figure S5D). Consistent with these findings, *ex vivo* isolated B cells from inflamed WAT of *Wipf1^−/−^* mice exhibited increased mRNA expression of *Ldha*, the enzyme responsible for converting pyruvate into lactate (Figure 5C), as well as the hypoxia marker *Hif1a* (Figure 5D). These results collectively support the notion of enhanced lactate secretion and a hypoxic, activated state in B cells within IBD-associated WAT, in agreement with our *in vitro* observations.

**Figure 5.**
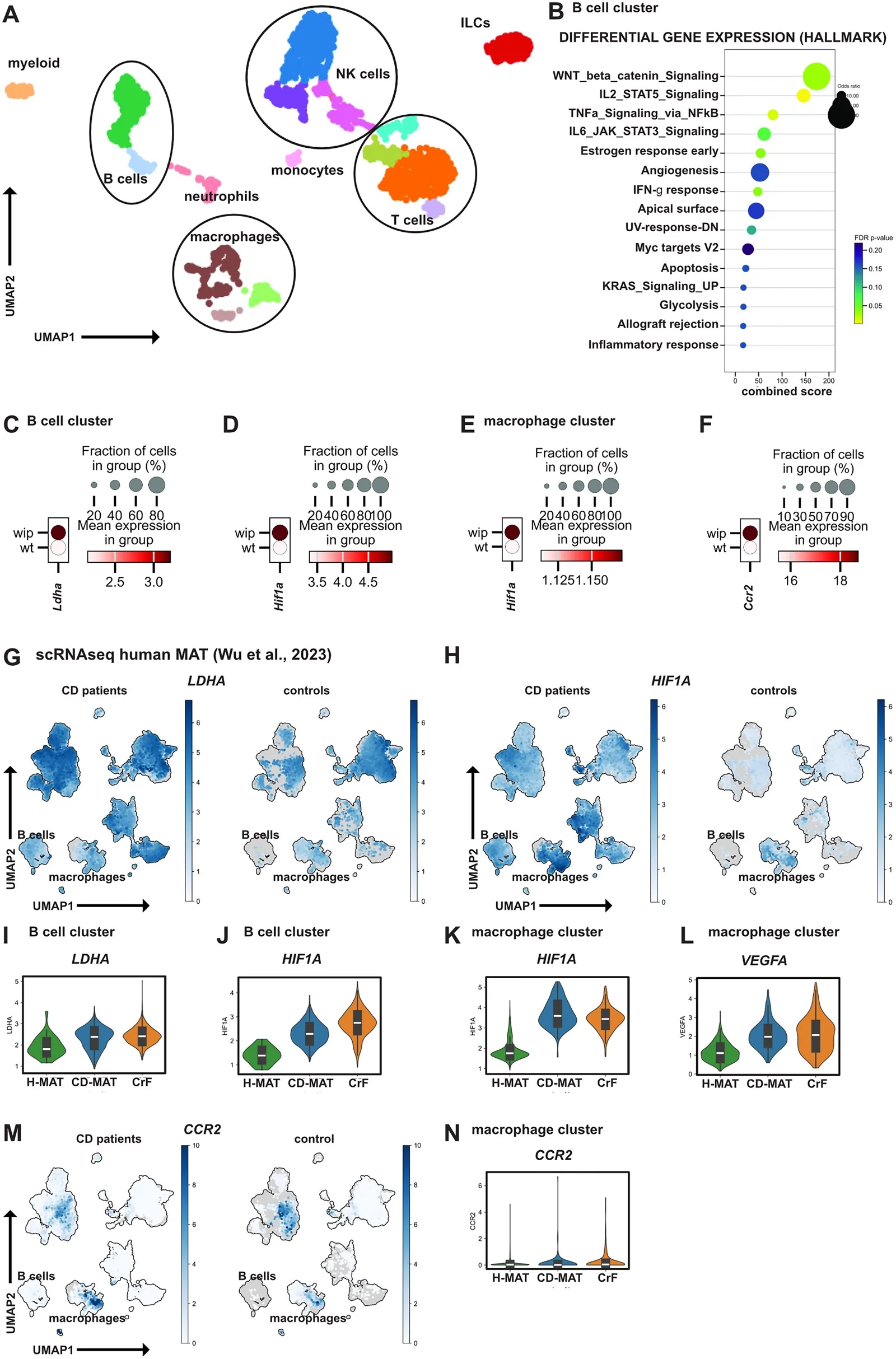
scRNAseq reveals increased LDHA and HIF1A mRNA in mouse IBD WAT and human CrF and a possible CCL2/CCR2-driven macrophage recruitment. (A) UMAP-representation of immune-cell clusters identified using scRNAseq analysis of CD45^+^ leukocytes isolated from gWAT of WT and *Wipf1^−/−^* mice. (B) Overrepresentation analysis of functional terms (Hallmark gene sets) in B cell clusters. Dot size reflects odds ratio, color shows FDR-adjusted *P*-value, and *x*-axis displays combined overrepresentation score. (C + D) mRNA expression levels of indicated genes in the B cell clusters split by genotype. Dot size indicates the proportion of cells expressing each gene within the group, while color intensity reflects the mean expression level. (E + F) Dot plot illustrating mRNA expression levels of indicated genes in the macrophage clusters split by genotype. Dot size indicates the proportion of cells expressing each gene within the group, while color intensity reflects the mean expression level. (A–F) *n* ≥ 2 mice per genotype. (G + H, M) UMAP representation of expression levels of (G) *LDHA*, (H) *HIF1A*, and (M) *CCR2* in CrF/MAT of CD patients compared to control MAT (data extracted from Wu et al. 2023, *n* = 2 HD MAT, *n* = 3 CD MAT, *n* = 6 CrF [three paired with CD MAT]). (I–L, N) Violin plots showing the distribution of (I) *LDHA*, (J + K) *HIF1A*, (L) *VEGFA*, and (N) *CCR2* between CrF and uninvolved MAT (CD-MAT) (three paired samples) and MAT from healthy donor controls (H-MAT) in the indicated clusters (data extracted from Wu et al. 2023, *n* = 2 HD MAT, *n* = 3 CD MAT, *n* = 6 CrF [three paired with CD MAT]).

Applying DGE analysis (hallmark gene set) to the macrophage cluster likewise demonstrated significant enrichment of transcripts related to inflammation, pro-inflammatory cytokine response, and hypoxia in macrophages isolated from inflamed gWAT of *Wipf1^−/−^* mice (Figure S5E). In line with these findings, we detected strong expression of *Hif1a* in macrophages from adipose tissue of *Wipf1^−/−^* mice (Figure 5E). Transcription factor activity analysis revealed a heightened *Hif1a* signature in these macrophages (Figure S5F), and pathway activity inference indicated increased hypoxia-related signaling (Figure S5G). Notably, pathways downstream of HIF-1α associated with M2-like macrophage polarization, such as *Vegfa*, were also upregulated (Figure S5G), suggesting a macrophage response tailored to a hypoxic and inflamed milieu. Consistent with our *in vitro* data, macrophages from WAT of *Wipf1^−/−^* mice displayed increased expression of *Arginase1* (Figure S5H), and together with observed CD206 expression (Figure 2E + F), this points toward an M2-like phenotype. Furthermore, these macrophages exhibited higher mRNA levels of the C-C chemokine receptor 2 (*Ccr2*), indicating an enhanced capacity to migrate along MCP-1 gradients (Figure 5F). Overall, these findings provide robust evidence for the accumulation of M2-like macrophages with an inflammatory signature under hypoxic conditions within inflamed adipose tissue of *Wipf1^−/−^* mice, likely driven by the interplay of B cell/adipocyte crosstalk and MCP-1-mediated recruitment.

To further elucidate the inflammatory microenvironment of CD-associated adipose tissue, we re-analyzed an scRNAseq dataset that compared CrF with uninvolved mesenteric adipose tissue from CD patients (CD-MAT), as well as mesenteric adipose tissue from healthy controls (H-MAT) (Figure 5G–N, Figure S6A–C).^6^ UMAP analysis of these datasets revealed a general increase in mRNA expression levels of *HIF1A* and *LDHA* within the MAT of CD patients (Figure 5G + H). This pattern mirrors our previous observations in IBD-associated mouse WAT. B cells isolated from the CrF of CD patients exhibited higher mRNA expression levels of both *LDHA* and *HIF1A* compared to B cells from control MAT (Figure 5I + J). Additionally, macrophage clusters within these datasets showed elevated levels of *HIF1A* and *VEGFA* (Figure 5K + L), indicating a shared hypoxic and pro-angiogenic signature. We also observed that CCR2 mRNA expression was predominantly upregulated in the monocyte/macrophage clusters of MAT from CD patients (Figure 5M + N), closely resembling our findings in inflamed mouse WAT. Notably, in this human dataset, mesenchymal stromal cells and other non-immune cell populations displayed increased mRNA levels of MCP-1 (*CCL2*) (Figure S6C). This observation, as characterized in greater detail by the original publication,^6^ supports our *in vitro* findings showing MCP-1 secretion by CrF-derived adipocyte spheroids (Figure 1K).

Collectively, our analyses of scRNAseq data from both mouse IBD-associated WAT and human CrF reinforce the hypothesis that B cell/adipocyte crosstalk drives the formation of a lactate-rich, hypoxic, and inflammatory microenvironment. This is characterized by enhanced MCP-1 production, which likely facilitates the recruitment and polarization of macrophages, and may consequently contribute to the progression of IBD-associated WAT inflammation.

## 4. Discussion

Here we investigated how B cells shape the inflammatory responses in IBD-associated adipose tissue. We used high-parametric flow cytometry and showed an accumulation of B cells in human and mouse IBD-associated adipose tissue. Co-transferring B cells in the T cell transfer colitis model enhanced TNF-α secretion by CD4 T cells and macrophage accumulation in mesenteric WAT. Depleting B cells through anti-CD20 antibody treatment diminished T cells and M2-like macrophage accumulation in WAT in a mouse model of IBD. Using *in vitro* co-cultures, we found that B cell/adipocyte crosstalk not only resulted in enhanced B cell activation but also led to elevated cytokine secretion by primary adipocytes, especially of MCP-1. This was in part due to the presence of TNF-α in the culture system. Of note, supernatants of the B cell/adipocyte co-culture promoted M2-like macrophage polarization *in vitro*, which correlated with enhanced levels of lactate. Concomitantly, scRNAseq analysis revealed that *ex vivo* isolated B cells from IBD-associated WAT of *Wipf1^−/−^* mice as well as from CrF of CD patients demonstrated enhanced mRNA levels of *Ldha*, indicating lactate production also *in vivo*. Interestingly, LPS-stimulated adipocyte spheroids *in vitro*, as well as macrophages and B cells isolated from human or mouse *ex vivo*, displayed a hypoxic signature, pointing at a hypoxic environment in inflamed WAT. We thus hypothesize that the presence of B cells in IBD-associated WAT contributes to enhanced MCP-1 secretion by adipocytes as well as the concentration of lactate in inflamed tissue. Elevated levels of MCP-1 together with the lactate-rich, hypoxic environment might accumulate and polarize M2-like macrophages. Taken together, our results provide evidence for a crucial role of B cell/adipocyte crosstalk to IBD-associated WAT inflammation.

While WAT inflammation related to obesity and aging has been well studied, CD-associated CrF remains a poorly understood phenomenon. Hyperplasia of CrF in CD patients or inflamed WAT of experimental models is characterized by smaller, but increased, numbers of adipocytes, as well as changes in their lipid metabolism.^4^^,^^6^^,^^7^^,^^31^ Using mid-infrared optoacoustic microscopy (MiROM), we recently found differences in the spectral distribution of adipocytes in IBD-related inflamed WAT of *Wipf1^−/−^* mice compared to WT tissue,^5^ also hinting at a difference in the lipid composition. Concomitantly, inflamed IBD-associated WAT, which contained both, adipocytes as well as immune cells, has been shown to express higher mRNA levels of inflammatory markers as well as chemoattractant molecules.^32^^,^^33^ To better characterize adipocyte inflammation without the contribution of immune cells, here we investigated two different *in vitro* 3D culture methods of human or mouse adipocytes. We examined primary isolated mouse mature adipocytes in transwell cultures and developed human and mouse adipose tissue-derived 3D spheroid cultures. In contrast to the often used 3T3 pre-adipocyte model, those *in vitro* cultures have been shown to exhibit and maintain characteristic features (eg, lipid metabolism) of *ex vivo* isolated WAT *in vitro*.^34^^,^^35^ Of note, we found that adipocyte tissue-derived spheroids from CrF of CD patients readily secreted enhanced levels of pro-inflammatory cytokines without additional LPS stimulation, despite the prolonged culture condition. Furthermore, LPS stimulation of human and mouse adipose tissue-derived spheroids mimicked the cytokine secretion profile of unstimulated CrF-derived spheroids in our *in vitro* cultures. This is to the best of our knowledge the first characterization of a model of the pro-inflammatory potential of CrF adipocytes *in vitro*.

Adipocytes isolated from CrF patients retained an altered inflammatory cytokine-secretion phenotype after removal from their original tissue environment, indicating persistence of an inflammation-associated cellular state *ex vivo*. Such persistence is compatible with the broader concept of inflammatory memory and suggests epigenetic imprinting, although our data do not establish the molecular mechanism responsible for maintaining this state. Interestingly, during obesity, epigenetic regulation of adipogenesis as well as epigenetic transgenerational inheritance has been described.^36^ In CD, recent evidence pointed at metabolically primed fibroblasts isolated from stricturing CD compared to non-stricturing CD, correlating with distinct histone deacetylase expression profiles.^37^ Thus, pre-adipocytes isolated from the SVF of CrF of CD patients might be epigenetically primed to secrete pro-inflammatory cytokines, which might play a role in the recurrence of intestinal inflammation after bowel resection in CD patients. However, few studies have investigated the influence of mesenteric removal on postoperative recurrence in CD patients.^38^^,^^39^

Although prominently present in CrF of CD patients, the role of B cells in CD-associated inflamed WAT has not been addressed so far. In contrast, during obesity or aging, B cells have been demonstrated to be amongst the first immune cells to accumulate in inflamed WAT,^40^ and IgG antibodies have been demonstrated to be sufficient to cause insulin resistance in diet-induced obesity. Consequently, the absence of B cells and IgG antibodies, either by B cell depletion therapy or using B cell-deficient mice, prevented adipose tissue inflammation, WAT fibrosis, and insulin resistance in diet-induced obesity or aging.^13^^,^^14^^,^^41^^,^^42^ Our findings additionally highlight a central role of B cells in IBD-associated, non-obese, non-aged WAT inflammation. Our *in vitro* data indicate that activated B cells promote MCP-1 secretion by primary mature, LPS-stimulated adipocytes through soluble mediators. In visceral WAT, MCP-1 production by stromal cells has been shown to depend on TNF-α derived predominantly from myeloid cells.^43^^,^^44^ Our findings raise the possibility that activated B cells may provide an additional source of inflammatory signals (including TNF-α) within WAT that stimulates adipocytes to produce MCP-1 and thereby promote the recruitment and polarization of M2-like macrophages. The soluble factors present in the B cell/adipocyte co-culture conditioned medium, including IgG antibodies, IL-10, other metabolites, or short-chain fatty acids, may contribute to macrophage polarization and remain to be defined.

B cell/adipocyte co-culture also increased CD86 expression on activated B cells. We previously identified CD86 as a key driver of pathogenic CD4 T-cell activation in *Wipf1^−/−^* mice, and blockade of CD86-mediated co-stimulation with CTLA-4-Ig attenuated intestinal inflammation.^24^ Consistent with these findings, co-transfer of B cells in the acute T cell transfer colitis model increased the frequency of TNF-α-producing CD4 T cells in WAT. Together, these observations suggest that activated B cells may amplify adipose tissue inflammation through two complementary mechanisms: by directly promoting adipocyte MCP-1 production and by enhancing CD4 T-cell activation via CD86-dependent co-stimulation, thereby increasing local TNF-α availability. The magnitude of these effects may depend on the activation state of the B cells, with *Wipf1^−/−^* B cells representing an amplified inflammatory model. Further studies are required to define the molecular mediators of B cell–adipocyte and B cell/T cell crosstalk in mouse and human IBD-associated WAT.

Inflamed, IBD-associated adipose tissue has been linked to a hypoxic response, characterized by enhanced expression levels of *HIF1A* and lactate.^7^ In hypoxic conditions, cells increasingly switch to anerobic glycolysis and convert pyruvate into lactate rather than entering the citric acid cycle after its conversion into acetyl-CoA. In addition, activated adipocytes also internally increase their oxygen consumption, leading to relative adipocyte hypoxia and lactate production.^45^^,^^46^ Using targeted metabolomics of culture supernatants of LPS-stimulated adipose tissue-derived spheroids, we detected increased levels of a hypoxic marker, orotidine, as well as extracellular lactate and diminished citrate levels. Furthermore, we found enhanced mRNA expression levels of *Hif1a* in LPS-stimulated, adipose tissue-derived spheroids, pointing at a hypoxic response in ambient oxygen conditions in a cell culture incubator. Thus, our 3D cell cultures suggested that adipose tissue hypoxia in IBD-associated, inflamed WAT might be initiated by adipocytes reacting to the presence of bacteria after barrier disruption. In addition, B cells might also contribute to enhanced local lactate concentrations in human and mouse inflamed IBD-associated adipose tissue.

The effects of lactate on immune cells seem to be highly cell- and context-dependent.^47^ Lactate has been shown to promote the differentiation of anti-inflammatory, pro-angiogenic M2-like macrophages.^29^^,^^30^ During aging, lactate seems to support B cell differentiation into age-associated B cells, characterized by autoantibody production, and the secretion of a senescence-associated secretory profile, such as IL-6 and TNF-α.^48^ In addition, lactate regulates the formation of ectopic lymphoid follicles during Sjoegren’s disease.^49^ Thus, lactate might also influence B cell differentiation, and immune cell accumulation and activation in IBD-related inflamed adipose tissue. We cannot exclude that a lactate-rich milieu influences B cell survival and IgG plasmablast differentiation in our *in vitro* assays. Experiments are under way to address the exact mechanisms of how adipocytes promote B cell survival and influence B cell activation.

Several limitations of our study should be acknowledged. First, the heterogeneity of treatment regimens in our human cohort, including the use of biologics, represents a potential confounding factor. Although we did not observe apparent treatment-associated differences in isolated cell populations or pre-adipocyte cytokine secretion, an influence of medication cannot be fully excluded. Second, given the substantial tissue- and disease-specific heterogeneity of macrophages, our characterization of M2-like macrophages was based primarily on CD206 expression,^1^ following the nomenclature established for CrF in CD. Additional phenotypic and functional analyses will be required to more comprehensively define this potentially disease-associated macrophage population. Finally, while our data support an interplay between B cells, adipocytes, inflammatory mediators, and metabolic changes, the individual causal relationships, including the contribution of B cell-derived TNF-α to MCP-1 induction, remain to be established.

The chemoattractant MCP-1 is a key player in attracting monocytes/macrophages to inflamed obese or aged adipose tissue or IBD-associated WAT inflammation.^33^^,^^50-52^ In addition, serum MCP-1 levels are associated with chronic inflammatory conditions during aging, obesity, and autoimmune diseases. Of note, in UC patients, reduced serum levels of MCP-1 have been associated with successful therapy responses using anti-TNF-α antibodies.^53^ Recent data suggested a high and specific expression of the MCP-1 gene in a cluster with other fibrosis-associated genes as a genome-wide association signal for CD.^6^ Evidence from our re-analysis of this scRNAseq dataset from CD patients together with results from our *in vitro* and *in vivo* studies extend these findings by suggesting that an initial infiltration of B cells into adipose tissue might exacerbate the secretion of MCP-1 by adipocytes and contribute to a lactate-rich environment. In addition to the presence of specific bacteria, the inflammatory, hypoxic, and lactate-rich IBD-related adipose tissue microenvironment likely supports pro-fibrotic, M2-like macrophage polarization. We recently described that depleting B cells through anti-CD20 antibody treatment at the onset of disease diminished intestinal inflammation in a mouse model of IBD.^24^ Our findings described here indicate that early B cell depletion might prevent or delay M2-like macrophage accumulation and reduces CD4 T cell cytokine secretion, and thus the propagation of the vicious circle of IBD-related adipose tissue inflammation. Overall, our data suggest that the modulation of B cell activation during intestinal and adipose tissue inflammation might add to the therapeutic options for the treatment of IBD. Following the recent successes of B cell depletion therapy using CD19 CAR T cells in treating autoimmune diseases,^54-56^ including IBD,^57^ here we present additional evidence supporting the key role of B cells in promoting inflammation across various types of tissues.

## Supplementary Material

jjag182_Supplementary_Data

## Data Availability

All data supporting the findings of this study are available within the article or the supplemental materials and from the corresponding author upon reasonable request. Raw scRNA-seq data (FASTQ format) are available from the Annotare repository (EMBL-EBI, accession number: E-MTAB-17384).
