# Supplementary material for: B cell/adipocyte crosstalk amplifies MCP-1 secretion, M2-like macrophage accumulation, and polarization in gut-associated, inflamed adipose tissue
Source: J Crohns Colitis. 2026 Nov 7;20(11):jjag182. doi: 10.1093/ecco-jcc/jjag182 (PMC13744403; doi:10.1093/ecco-jcc/jjag182)
Supplement: Supplementary file 1 [file jjag182_supplementary_data.pdf]

Supplemental Files

B cell/adipocyte crosstalk amplifies MCP-1 secretion, M2-like macrophage accumulation and polarisation in gut-associated, inflamed adipose tissue

Authors:

Marie Christine Goess[1,2], Julia M. Heinze[1,2], Julian Hofmann[3], Annalisa Buck[4,5], Iana Gadjalova[1,2], Marie-Christin Weber[4], Sandra Ring[1,2], Joerg M. Buescher[6], Matteo Villa[7], Philipp-Alexander Neumann[4,5], Jürgen Ruland[1,2,8,9], Selina J. Keppler[1,2,7*]

Supplemental Figures

Supplemental Figure 1 – related to Figure 1

Supplemental Figure 2 – related to Figure 2

Supplemental Figure 3 – related to Figure 2

Supplemental Figure 4 – related to Figure 3

Supplemental Figure 5 – related to Figure 5

Supplemental Figure 6 – related to Figure 5

Supplemental Tables

Supplemental Table 1: patients` characteristics

Supplemental Table 2: list of antibodies

Supplemental Table 3: list of oligonucleotides

Supplemental Material and Methods

Suppl. Fig. 1 related to Fig. 1

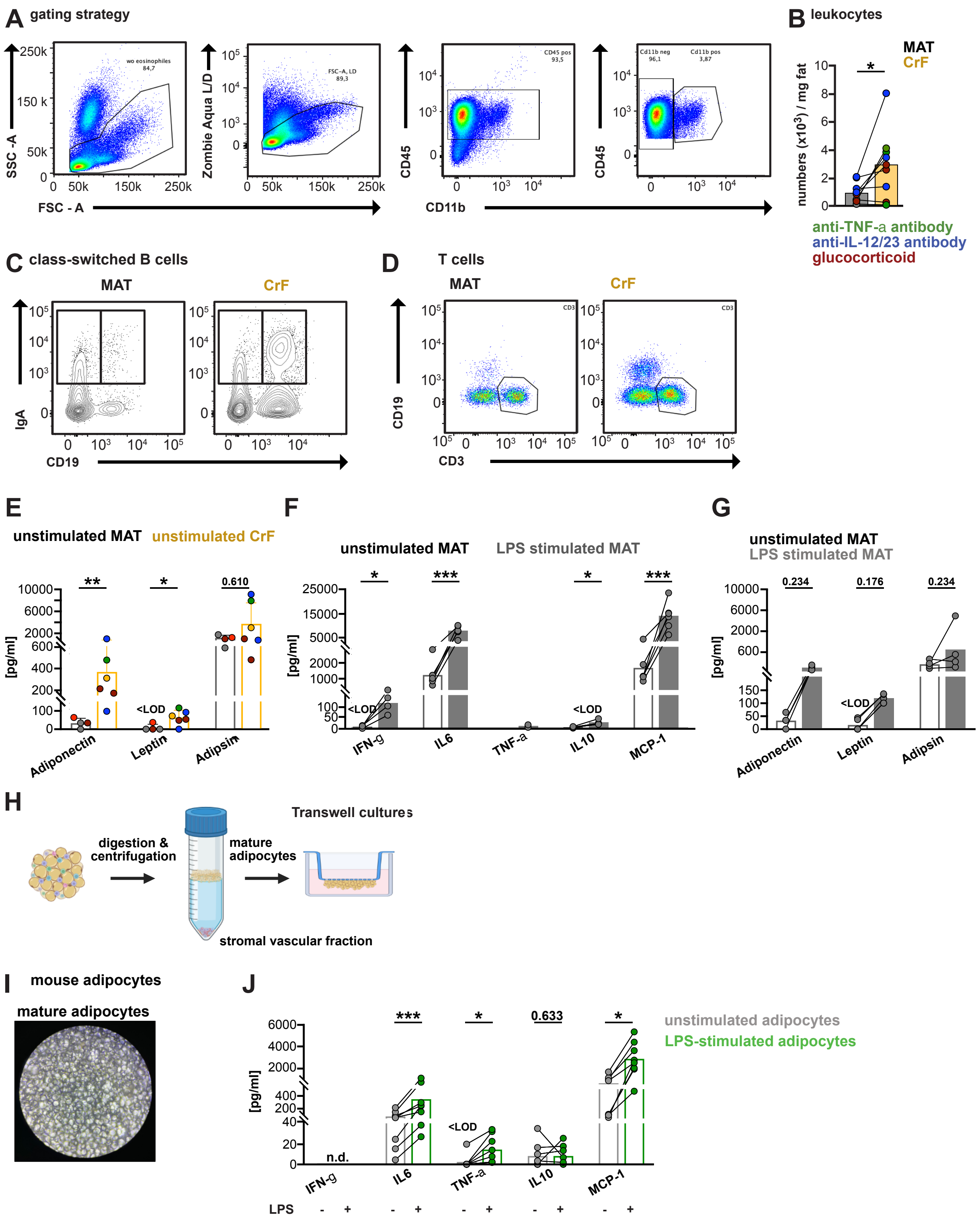

Suppl. Fig. 1:
(A) Gating strategy for leukocytes in single cell suspensions isolated from MAT and CrF of CD patients. (B) Total number of living leukocytes ($CD45^+$) per mg fat tissue from MAT and CrF. *Treatment indicated by colour coding.* (C+D) Representative plots of (C) class-switched B cells and (D) CD3+ T cells, gated on $CD45^+$, $CD11b^-$. (E) Adipokine secretion profile in the supernatants of differentiated MAT- or CrF-derived adipocyte spheroids was measured using a multiplex assay (mean ± SD). Treatment indicated by colour coding. (F) Cytokine and (G) adipokine secretion pattern of differentiated MAT-derived adipocyte spheroids cultured under non-simulated and LPS-stimulated conditions was measured using a multiplex immunoassay (Legenplex) on collected culture supernatants. For comparison purposes, cytokines of supernatants of unstimulated MAT-derived spheroids plotted are the same in Fig. 1K and Suppl. Fig. 1F. *Spheroids were generated from n≥5 patients. Quantification is shown as mean.* (H) Experimental setup of the adipose transwell culture system. (I) Image of murine primary mature adipocytes isolated from gWAT of WT mice cultured under a permeable transwell membrane (10x magnification). (J) Cytokine secretion profile of murine mature adipocytes under non-simulated and LPS-simulated conditions was measured using a multiplex immunoassay on transwell culture supernatants. *Transwell cultures were generated from n≥8 WT mice, pooled from 4 independent experiments. Quantification is shown as mean. Statistical significance was calculated using (B) Wilcoxon signed-rank test, (E) Mann-Whitney test, (F-J) paired t-test or Wilcoxon-signed rank test followed by Holm-Sidak correction as specified in the methods section. *p < 0.05; **p < 0.01; ***p < 0.001; ****p < 0.0001. Illustration 1H was created withBioRender.com.*

Suppl. Fig. 2 related to Fig. 2

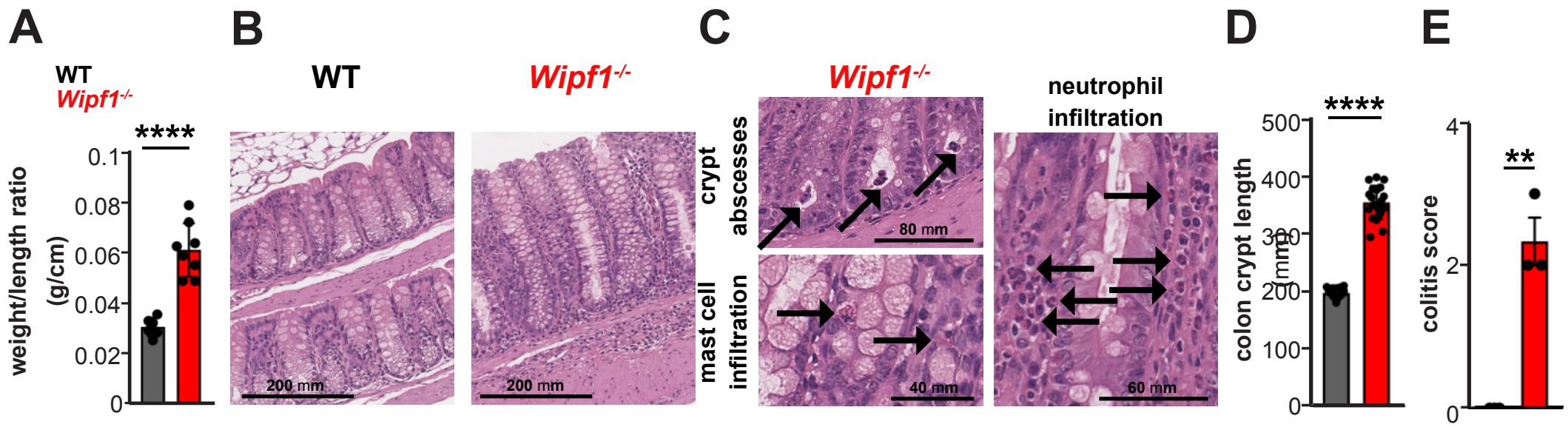

F

Inflammatorycell infiltrate		epithelial changes	mucosalarchitecture	Score
severity	extent			
Minimal	Mucosa	Minimal hyperplasia		1
Mild	Mucosa, sometimessubmucosa	Mild hyperplasia,mild goblet cell loss±cryptitis± erosions		2
Moderate	Mucosa and submucosa	Moderate hyperplasia, moderate goblet cell loss,cryptitis± crypt abscesses	Ulcerations	3
Marked	Mucosaand submucosa,often transmural	Markedhyperplasia, markedgoblet cell loss,multiple crypt abscesses	Extendedulcerations± pseudopolyps	4

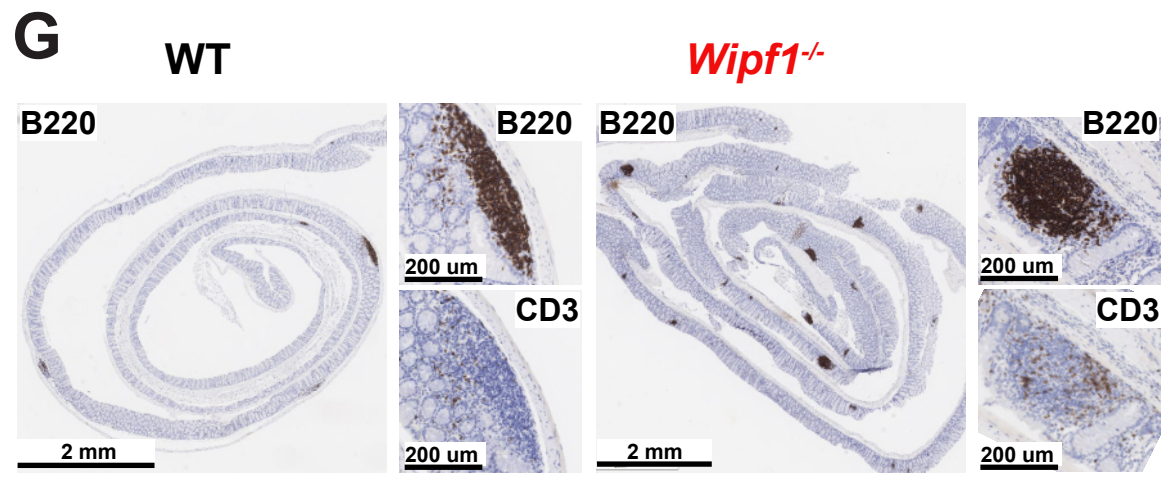

Suppl. Fig. 2: Reproduced and adapted from Figure 1 from Gadjalova I, Heinze JM, Goess MC, et al. *Mucosal Immunology*. 2024;17:67–80, under the terms of the Creative Commons Attribution 4.0 International (CC BY 4.0) licence. **(A)** Length and weight of the colon from WT and *Wipf1*$^{-/-}$ mice was measured and weight to length ratio calculated (n=8 per genotype, pooled from 4 independent experiments, mean ± SD) Statistical significance was calculated using student`s t test. **(B)** Representative pictures of colonic sections of WT or *Wipf1*$^{-/-}$ mice stained for H&E. **(C)** Arrows in zoom-ins of colonic sections of *Wipf1*$^{-/-}$ mice demonstrate crypt abscesses, mast cell infiltration and neutrophil infiltration.**(D)** Colonic crypt length measured from sections stained with H&E (total of 3 images, 20 crypts each, mean ± SD). Statistical significance was calculated using student`s t test. **(E+F)** Colonic inflammation in *Wipf1*$^{-/-}$ mice was scored according to the Table **(F)**(*16)*.
(G) Immunohistochemistry of sections of colonic swiss rolls stained for B220. Zoom-ins of isolated lymphoid follicles (ILFs) indicating B220 as well as CD3 staining.

Suppl. Fig. 3 related to Fig. 2

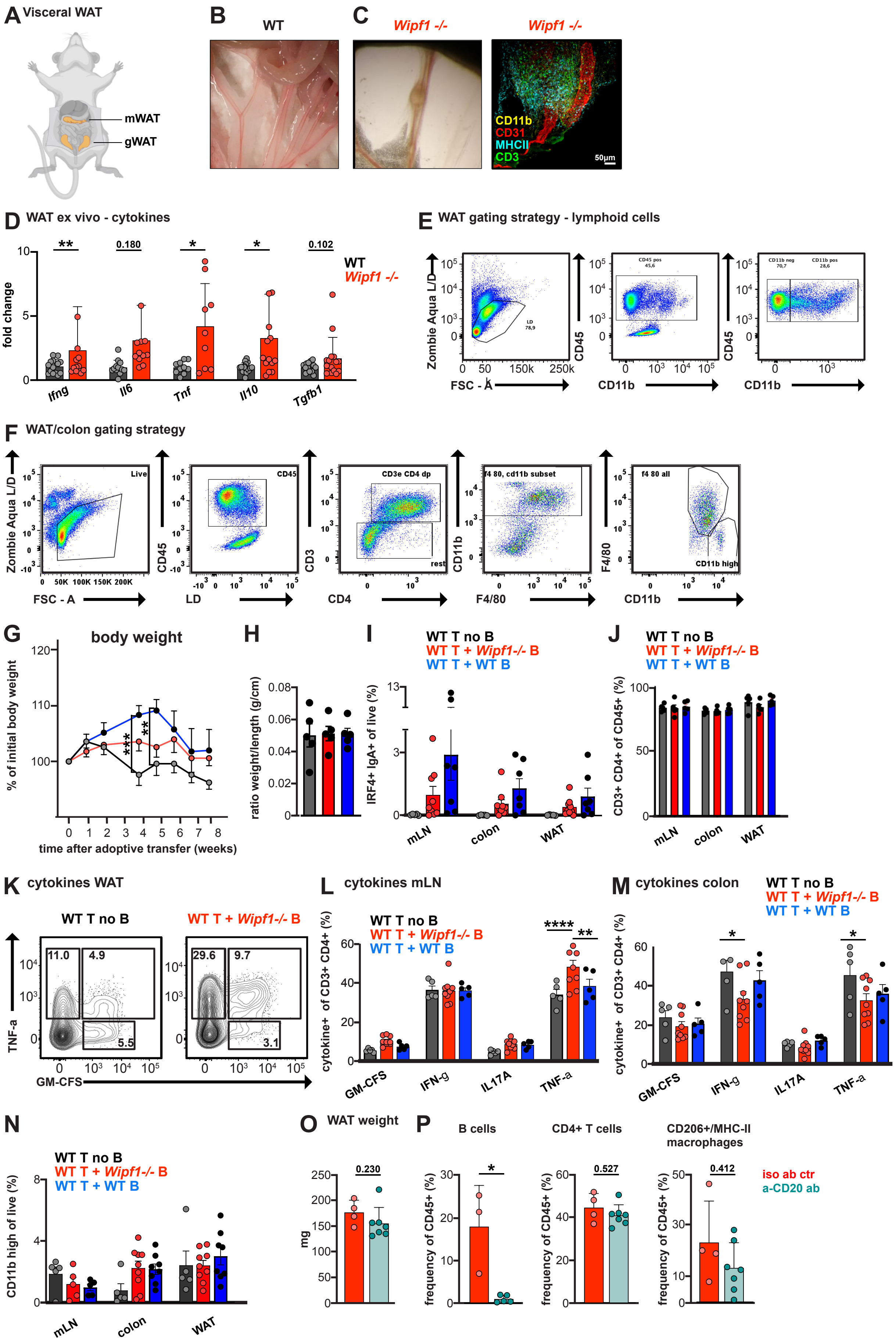

Suppl. Fig. 3:
(A) Schematic depicting the regions of visceral white adipose tissue (mWAT and gWAT) in mice collected for analysis. (B) Micrograph image of mWAT of a WT mouse. (C) Micrograph and immunofluorescent image of a lymphoid structure in mWAT of Wipf1$^{-/-}$ mice. (D) Relative mRNA expression of cytokines in ex vivo isolated mWAT from WT and Wipf1$^{-/-}$ mice. *n ≥ 15 per genotype, pooled from 5 independent experiments. Statistical significance was determined using an unpaired student´s t test.* (E+F) Flow cytometry gating strategy used to assess the frequencies of cells in single cell suspensions prepared from visceral WAT. (G) Body weight of *Rag1$^{-/-}$* recipient mice in the T cell transfer colitis model shown as percentage of starting weight. (H) Colon weight to length ratio of Rag1$^{-/-}$ recipient mice. (I) Frequencies of IgA^{+} antibody-secreting cells isolated from mLN, colonic lamina propria, and mWAT. (J) Frequencies of CD3^{+},CD4^{+} T cells isolated from mLNs, colonic tissue or mWAT. (K) Representative flow plots of CD3^{+},CD4^{+} T cells isolated from mWAT showing intracellular expression of indicated cytokines. (L+M) Frequencies of CD3^{+},CD4^{+} T cells isolated from (L) mLN and (M) colonic tissue expressing indicated cytokines intracellularly after in vitro re-stimulation. (N) Frequencies of CD11b^{high} myeloid cells isolated from mLNs, colonic tissue or mWAT. *n ≥ 5 per genotype, pooled from 2 independent experiments. Statistical significance was calculated using ordinary two-way ANOVA with Tukey`s multiple comparison test. Only significant results are indicated.* (O) mWAT weight was recorded following tissue harvest from experimental mice. (P) Frequencies of B cells, T cells, and CD206^{+}/MHCII^{+} (M2-like) cells were measured in WAT following anti-CD20 antibody treatment. *n≥3 mice per genotype, pooled from 3 independent experiments. Statistical significance was determined using a Mann-Whitney test. *p < 0.05; **p < 0.01; ***p < 0.001; ****p < 0.0001.* Illustration 2A was created with BioRender.com.

Suppl. Fig. 4 - related to Fig. 3

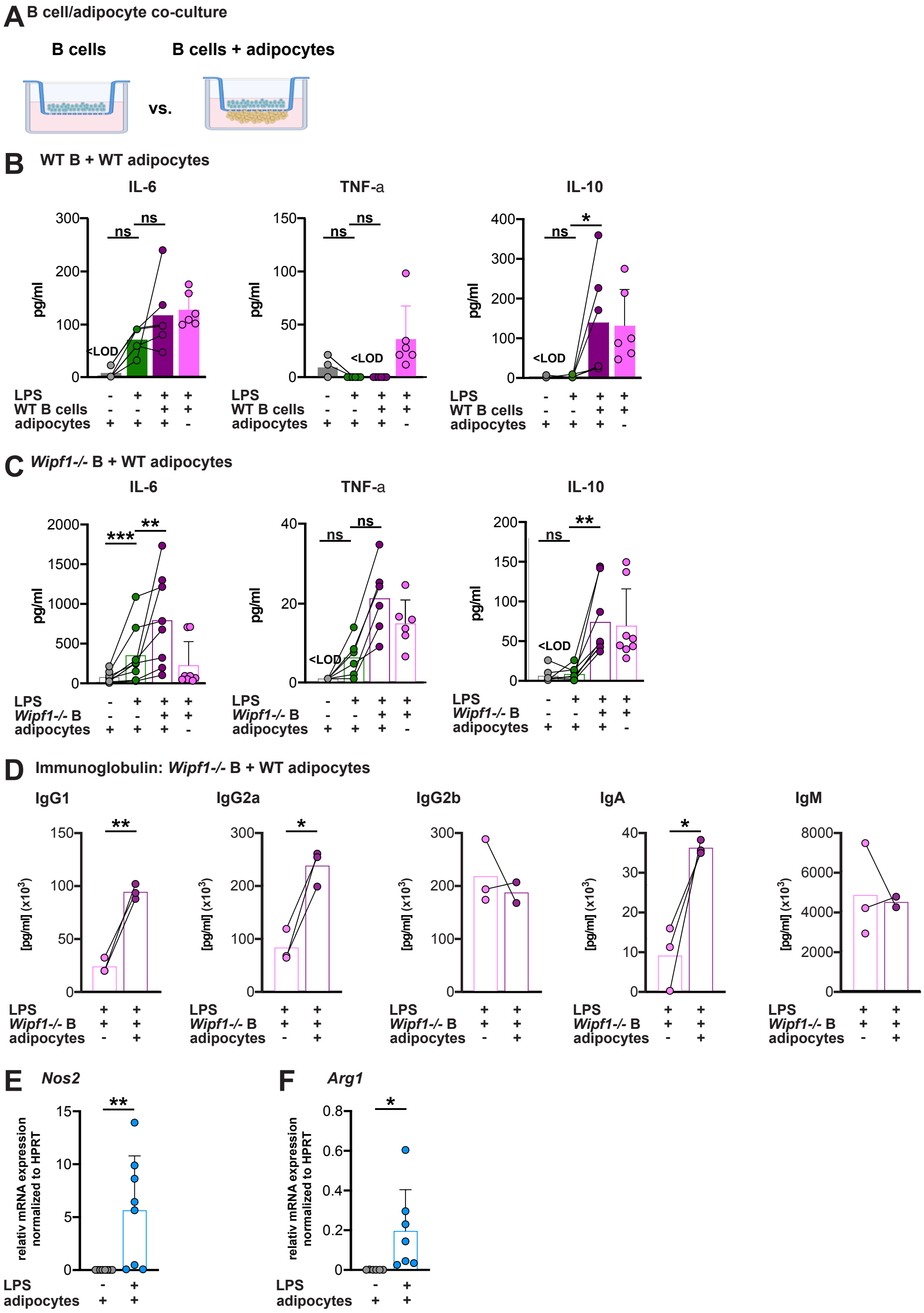

Suppl. Fig. 4:(A) Schematic representation of the experimental design for B cell/adipocyte transwell co-cultures. (B) Cytokine concentrations in the culture supernatants of WT B cells co-cultured with WT adipocytes. n=6 mice pooled from 2 independent experiments. (C) Cytokine concentrations in the culture supernatants of Wipf1-/- B cells co-cultured with WT adipocytes. n≥7 mice pooled from 4 independent experiments. (D) Concentrations of immunoglobulins released into the supernatants from mono- and co-cultures of stimulated Wipf1-/- B cells with adipocytes were measured using multiplex assays. Data is shown as mean, n≥2 mice. (E+F) Relative mRNA expression levels of (E) *Nos2* and (F) *Arg1* in BMDMs were measured after 24 hours of exposure to conditioned media as indicated. Results are shown as mean ± SD, n≥7 mice per condition pooled from 4 independent experiments. Statistical significance was calculated using (B+C) parametric analysis: one-way repeated measures ANOVA with Geisser-Greenhouse correction, followed by Holm-Sidak; or nonparametric analysis (<LOD): Friedman test followed by Dunn`s multiple-comparison test. *The B cell + LPS condition is included in the graph for descriptive comparison but was excluded from statistical testing as it is not part of the matched adipocyte comparison*; (D) paired student´s t test or (E,F) Wilcoxon-signed rank test. *p < 0.05; **p < 0.01; ***p < 0.001; ****p < 0.0001. Illustration 3A was created with BioRender. com.

Suppl. Fig. 5 - related to Fig. 5

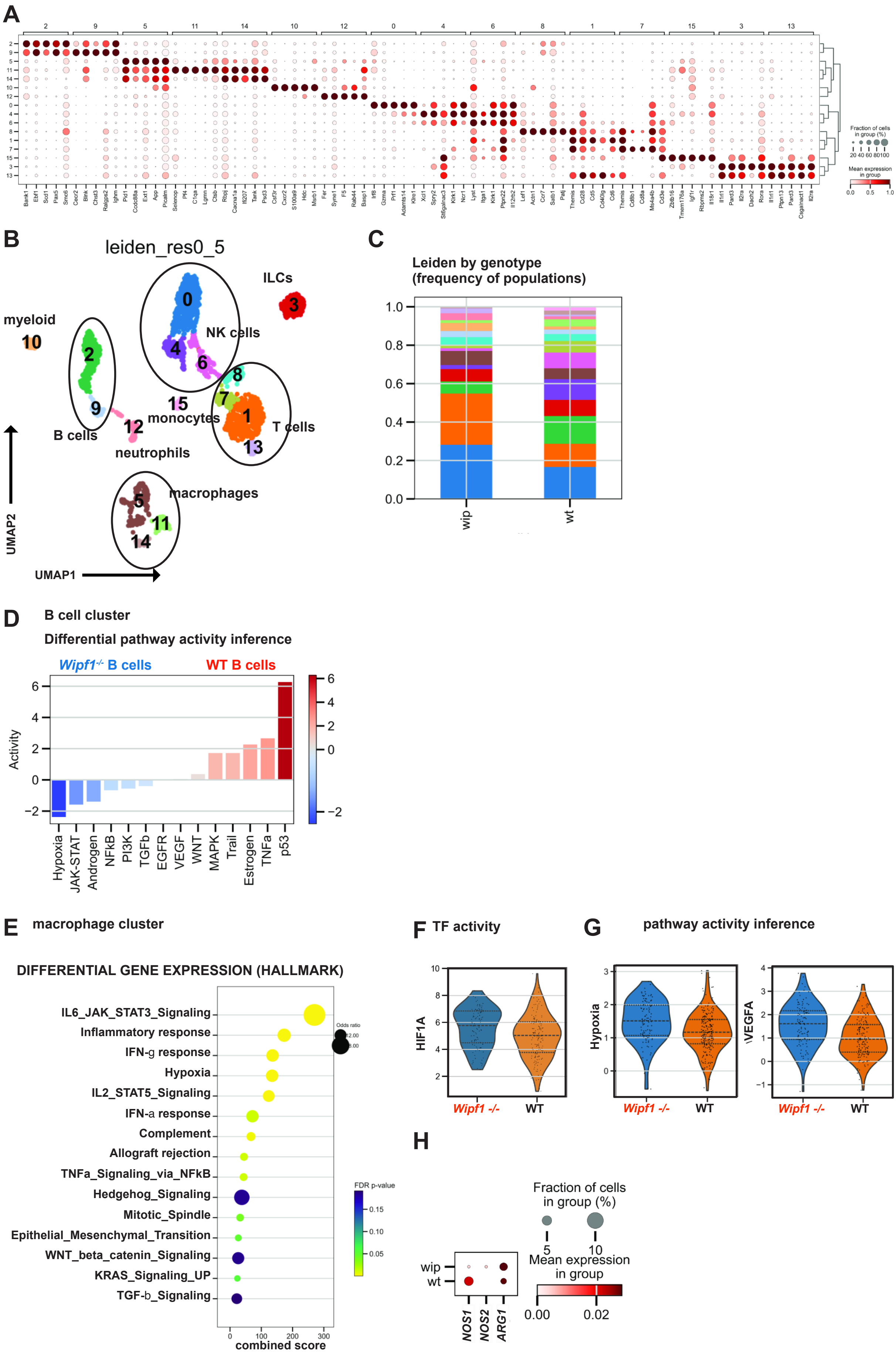

Suppl. Fig. 5:
(A) Dot plot showing the top five genes of each identified cell cluster most differentially expressed as identified by scanpy in single-cell RNA sequencing of CD45+ leukocytes isolated from murine gWAT. Dot size indicates the proportion of cells expressing each gene within a cluster, and color intensity represents the average expression level. (B) UMAP-representation of immune-cell clusters identified using scRNAseq analysis of $CD45^{+}$ leukocytes isolated from WAT of WT and *Wipf1*$^{-/-}$ mice. (C) Frequency of leiden cluster distribution between the genotypes (D) Bar chart showing the distribution of inferred activity of indicated pathways in B cells between the genotypes. (E) Overrepresentation analysis of functional terms (Hallmark gene sets) in pooled macrophage clusters. Dot size reflects odds ratio, color shows FDR-adjusted p-value, x-axis displays combined overrepresentation score.(F) Violin plots showing the distribution of inferred transcription factor activity of *HIF1A* in macrophages between the genotypes. (G) Violin plots showing the distribution of inferred hypoxia and *VEGFA* pathway activity in B cells between the genotypes. (H) mRNA expression levels of indicated genes focusing on the macrophage cluster. Dot size indicates the proportion of cells expressing each gene within the group, while color intensity reflects the average expression level. n≥2 mice per genotype.

Suppl. Fig. 6 - related to Fig. 5

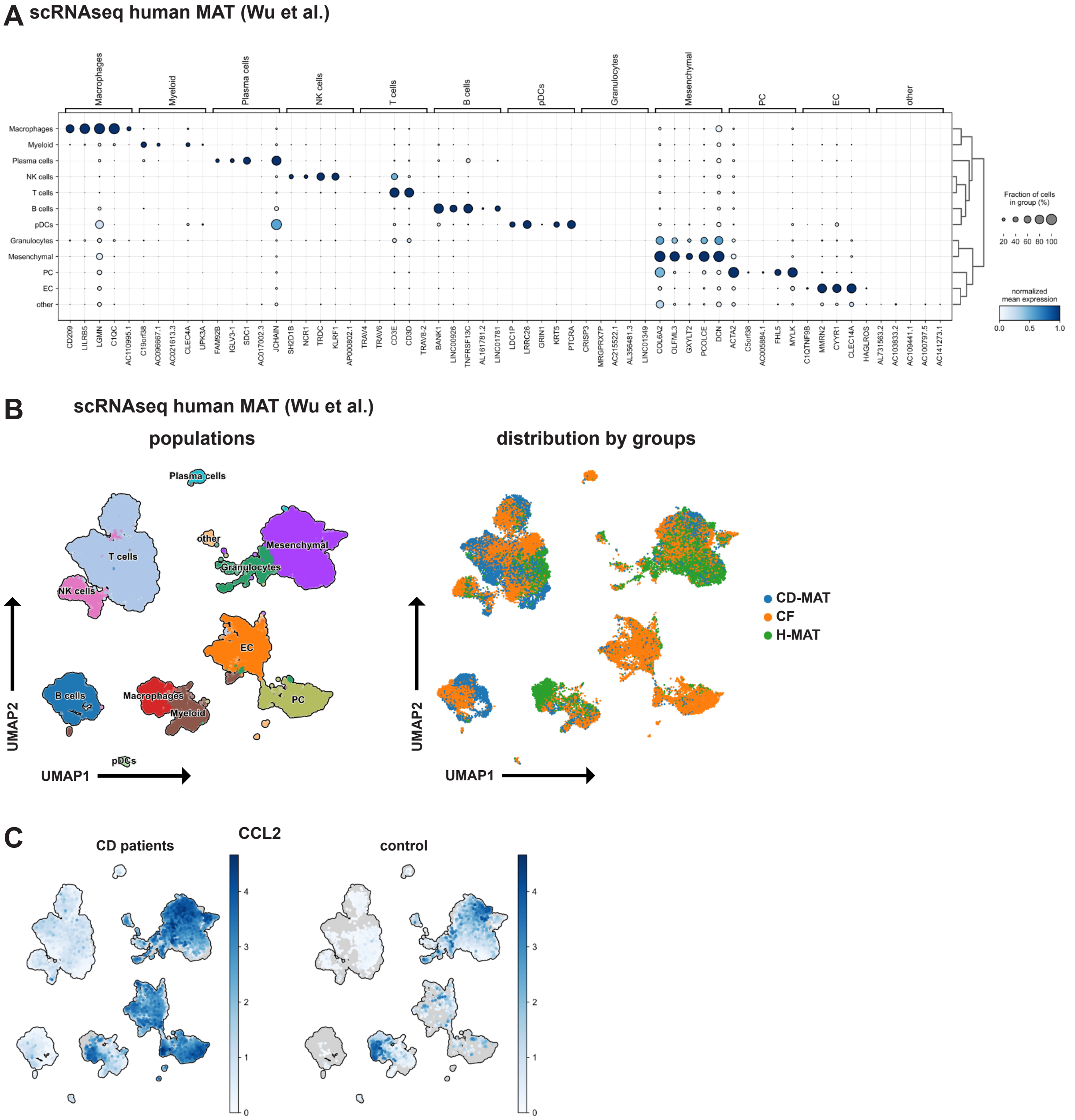

Suppl. Fig. 6:
(A)Dot plot showing the top five genes of each identified cell cluster most differentially expressed as identified by scanpy in single-cell RNA sequencing of single-cells isolated from HD-MAT, CD MAT and CrF (data extracted from (Wu et al., 2023)). Dot size indicates the proportion of cells expressing each gene within a cluster, and color intensity represents the average expression level. (B) UMAP-representation of cell clusters identified and the distribution by the different groups using scRNAseq analysis of single-cells isolated from HD-MAT, CD MAT and CrF. (C) UMAP-representation of expression levels of *CCL2* in CrF/MAT of CD patients compared to control MAT (n=2 HD MAT, n=3 CD MAT, n=6 CrF (3 paired with CD MAT).

Supplemental Table S1

**Patient ID**	**Age at resection**	**sex**	**diagnosis**	**year at first diagnosis**	**localization**	**sample**	**Medication at resection**	**flow cytometry?**	**spheroids?**
H9	31	m	Stricturing CD	2014	Terminal ileum	MAT/CrF	Adalimumab	Y	N
H13	24	m	Stricturing CD	2019	Terminal ileum	MAT/CrF	Glucocorticoide	Y	N
H16	55	f	Stricturing CD	1982	Terminal ileum	MAT/CrF	Ustekinumab	Y	N
H19	71	m	Stricturing CD	-	Terminal ileum	MAT/CrF	Ustekinumab	Y	N
H20	29	m	Stricturing CD	2016	Terminal ileum	MAT/CrF	Ustekinumab	Y	N
25	62	f	Stricturing CD	2004	Terminal ileum	**MAT/CrF**	-	N	Y
26/H5	70	f	Stricturing CD	1991	Terminal ileum	**MAT**/CrF	Glucocorticoide	Y	Y
29/H4	42	m	Stricturing CD	2002	Ascending colon	**MAT**/CrF	Infliximab	Y	Y
31	57	f	Stricturing CD	1994	Terminal ileum	CrF	Steroids	N	Y
32/H7	50	f	Stricturing CD	2021	Transverse colon	**MAT**/CrF	Glucocorticoide	Y	Y
34	31	m	Stricturing CD	2020	Terminal ileum	MAT	Vedolizumab	N	Y
35	46	m	Stricturing CD	2001	Sigmoid colon	CrF	Ustekinumab	N	Y
37	21	m	Stricturing CD	2019	Terminal ileum	CrF	Glucocorticoide	N	Y
41	21	m	Stricturing CD	2008	Terminal ileum	CrF	Ustekinumab	N	Y
46	28	m	Stricturing CD	2016	Terminal ileum	MAT	Ustekinumab	N	Y
56/H15	54	m	Stricturing CD	1997	Terminal ileum	MAT/**CrF**	Infliximab	Y	Y
60	55	f	Stricturing CD	1991	Terminal ileum	CrF	Glucocorticoide	N	Y

Supplemental Table S2

Antibodies and Dyes

**Antibody (Clone)**	**Target species**	**Fluorophore**	**Company**
BODIPY™ FLC16			Thermo Fisher
Zombie Aqua™ Fixable Viability Dye			BioLegend
CD19 (HIB19)	human	APC-Cy7	BioLegend
CD20 (2H7)	human	FITC	BioLegend
CD206 (MMR)	human	FITC	BioLegend
CD3 (HIT3a)	human	BV605	BioLegend
CD3 (HIT3a)	human	BV785	BioLegend
CD4 (SK3)	human	BV605	BioLegend
CD45 (2D1)	human	PerCP-Cy5.5	BioLegend
HLA DP DQ DR (TU39)	human	AF647	BioLegend
HLA DP DQ DR (TU39)	human	BV421	BD Biosciences
IgA (polyclonal)	human	AF555	Southern biotech
Human TruStain FcX	human		BioLegend
B220/CD45R (RA3-6B2)	mouse	BUV395	BioLegend
B220/CD45R (RA3-6B2)	mouse	BV421	BioLegend
CD11b (M1/70)	mouse/human	AF700	BioLegend
CD163 (S15049I)	mouse	BV421	BioLegend
CD19 (6D5)	mouse	BV785	BioLegend
CD20 (sa275a11)	mouse	AF700	BioLegend
CD206 (C068C2)	mouse	AF647	BioLegend
CD3e (145-2C11)	mouse	PerCP-Cy5.5	BioLegend
CD4 (GK1.5)	mouse	BV605	BioLegend
CD45 (30-F11)	mouse	BUV395	BioLegend
CD86 (GL-1)	mouse	PerCP-Cy5.5	BioLegend
MHCII (M5/114.15.2)	mouse	APC-Cy7	BioLegend
MHCII (M5/114.15.2)	mouse	AF488	BioLegend
Purified CD16.2 (9E9)	mouse		BioLegend
Purified CD16/32 (93)	mouse		BioLegend
Ultra-LEAF™ Purified IFNγ	mouse		BioLegend
Ultra-LEAF™ Purified IL-10	mouse		BioLegend
Ultra-LEAF™ Purified IL-6	mouse		BioLegend
Ultra-LEAF™ Purified MCP-1	mouse		BioLegend
Ultra-LEAF™ Purified TNFα	mouse		BioLegend

Supplemental Table S3

Oligonucleotides

Primers were synthesized at Eurofins Genomics (Ebersberg, Germany).

murine qPCR Primer

**Enzymes for macrophage classification**	
iNOS_for	CTT TGC CAC GGA CGA GAC
iNOS_rev	TCA TTG TAC TCT GAG GGC TGA C
Arg-1_for	GGA ATC TGC ATG GGC AAC CTG TGT
Arg-1_rev	AGG GTC TAC GTC TCG CAA GCC
Control	
Hprt_for	AGCCTAAGATGAGCGCAAGT
Hprt_rev	TTACTAGGCAGATGGCCACA
**Hypoxia**	
HIFa_for	CCT GCA CTG AAT CAA GAG GTT GC
HIFa_rev	CCA TCA GAA GGA CTT GCT GGC T
NOX2_for	CCCTTTGGTACAGCCAGTGAAGAT
NOX2_rev	CAATCCCGGCTCCCACTAACATCA

Supplemental Methods

Histology, Immunohistochemistry, Whole-mount Immunofluorescence

For histological analysis, murine colons were flushed with ice-cold PBS, opened longitudinally, rolled, and fixed in 4% neutral buffered formalin for 48 h. Samples were dehydrated, paraffin-embedded, and sectioned at 2 µm using standard procedures. Sections were stained with hematoxylin and eosin (H&E) or subjected to immunohistochemical (IHC) staining using antibodies against CD3 (clone SP7, DCS CI597R0, 1:100) and B220 (clone RA3-6B2, BD 550286, 1:50). IHC was performed on a Leica BondRxm using the Polymer Refine Detection Kit with 3,3-DAB as chromogen. All slides were scanned at 40× using a Leica AT2 system and analyzed with ImageScope (12.4.0.7018). H&E sections were analysed and scored in an unbiased manner using the scoring scheme in Suppl.Fig.2F, and crypt length was measured for 20 crypts per mouse using ImageJ. For immunofluorescence, WAT was processed as previously described (18). Tissue was fixed (IC fixation buffer, Thermo Fisher FB001; 1 h, RT), blocked overnight (1% (v/v) FCS, 1% (v/v) mouse serum, and 0.3% (v/v) Triton X-100 in PBS) at 37°C, and stained in blocking buffer with BV421 anti-CD163, AF488 anti-MHC-II, AF594 anti-CD11b, and AF647 anti-CD206 (all 1:200) for 72 h at 37°C. After washing (0.2 % (v/v) Triton X-100 and 0.5 % (v/v) 1-thioglycerol in PBS; 24 h, 37°C), tissue was dehydrated in graded isopropanol (30% - 50% - 70% - 100%, Sigma-Aldrich) and cleared with ethyl cinnamate (Sigma-Aldrich #W243000). Imaging was performed using an inverted Leica TCS SP8 confocal microscope equipped with a white light laser and HyD photodetectors, utilizing

an HC PL APO CS2 20×/0.75 IMM objective at a zoom factor of 1 (Leica Microsystems). Image deconvolution was carried out with the LIGHTNING module of the Leica Application Suite X (Leica Microsystems). Subsequent image analysis and contrast adjustments were completed in Imaris (Bitplane, version 9.5.1). Immune cells were segmented by generating binary masks with the Surface Creation Wizard in Imaris, and quantitative data for these objects were exported for cell quantification.
